# Nationwide projections of heat- and cold-related mortality impacts under various climate change and population development scenarios in Switzerland

**DOI:** 10.1088/1748-9326/ace7e1

**Published:** 2023-08-11

**Authors:** Evan de Schrijver, Sidharth Sivaraj, Christoph C Raible, Oscar H Franco, Kai Chen, Ana M Vicedo-Cabrera

**Affiliations:** 1 Institute of Social and Preventive Medicine (ISPM), University of Bern, Bern, Switzerland; 2 Oeschger Center for Climate Change Research (OCCR), University of Bern, Bern, Switzerland; 3 Graduate School of Health Sciences (GHS), University of Bern, Bern, Switzerland; 4 Climate and Environmental Physics, Physics Institute, University of Bern, Bern, Switzerland; 5 Julius Center for Health Sciences and Primary Care, University of Utrecht Medical Center, Utrecht, The Netherlands; 6 Department of Environmental Health Sciences, Yale School of Public Health, New Haven, CT, United States of America; 7 Yale Center on Climate Change and Health, Yale School of Public Health, New Haven, CT, United States of America

**Keywords:** shared socioeconomic pathways, representative concentration pathways, mortality, nationwide analysis, distributed lag non-linear model, heat and cold

## Abstract

Climate change and progressive population development (i.e., ageing and changes in population size) are altering the temporal patterns of temperature-related mortality in Switzerland. However, limited evidence exists on how current trends in heat- and cold-related mortality would evolve in future decades under composite scenarios of global warming and population development. Moreover, the contribution of these drivers to future mortality impacts is not well-understood. Therefore, we aimed to project heat- and cold-related mortality in Switzerland under various combinations of emission and population development scenarios and to disentangle the contribution of each of these two drivers using high-resolution mortality and temperature data. We combined age-specific (<75 and ⩾75 years) temperature-mortality associations in each district in Switzerland (1990–2010), estimated through a two-stage time series analysis, with 2 km downscaled CMIP5 temperature data and population and mortality rate projections under two scenarios: RCP4.5/SSP2 and RCP8.5/SSP5. We derived heat and cold-related mortality for different warming targets (1.5 °C, 2.0 °C and 3.0 °C) using different emission and population development scenarios and compared this to the baseline period (1990–2010). Heat-related mortality is projected to increase from 312 (116; 510) in the 1990–2010 period to 1274 (537; 2284) annual deaths under 2.0 °C of warming (RCP4.5/SSP2) and to 1871 (791; 3284) under 3.0 °C of warming (RCP8.5/SSP5). Cold-related mortality will substantially increase from 4069 (1898; 6016) to 6558 (3223; 9589) annual deaths under 2.0 °C (RCP4.5/SSP2) and to 5997 (2951; 8759) under 3.0 °C (RCP8.5/SSP5). Moreover, while the increase in cold-related mortality is solely driven by population development, for heat, both components (i.e., changes in climate and population) have a similar contribution of around 50% to the projected heat-related mortality trends. In conclusion, our findings suggest that both heat- and cold-related mortality will substantially increase under all scenarios of climate change and population development in Switzerland. Population development will lead to an increase in cold-related mortality despite the decrease in cold temperature under warmer scenarios. Whereas the combination of the progressive warming of the climate and population development will substantially increase and exacerbate the total temperature-related mortality burden in Switzerland.

## 1 Introduction

Current literature suggests a strong association between non-optimal temperatures (i.e., heat and cold) with several health outcomes. In particular, 9.4% of the total global mortality burden can be attributed to non-optimal temperature with the majority of deaths attributable to cold (8.4%, versus 0.7% for heat) [1]. Besides mortality, other adverse health outcomes have also been associated with non-optimal temperatures, such as hospital admissions due to cardiovascular and respiratory diseases [2–4]. Also deteriorated mental health, reduced sleep quality and lower work productivity and economic costs have been associated with heat [5–9]. Altogether, exposure to heat and cold results every year in a substantial health burden and economic costs for society, and it is expected to be larger in the coming years as climate change progresses [10].

In 2022 the warming of the planet has reached a new milestone, with the global mean temperature being 1.2 °C above the average in preindustrial times [11, 12]. The progressive warming of the planet has increased the odds of extreme heat events that were previously rarely observed and were not possible without the influence of anthropogenic climate change [13–16]. The impacts of climate change on human health are already visible. A recent attribution study found that 37% of all heat-related deaths can be attributed to anthropogenic climate change [17]. And it is projected that heat-related health burden will further increase in the coming decades as climate change progresses [13, 14, 18–22]. However, most projections studies have been limited to specific regions or countries, and the only available quasi-global assessments focused on urban locations [19, 23–26]. Moreover, a few studies have included projections of cold-related mortality and considered future changes in population size and demographic structure [27–34]. Not accounting for changes in population development has been argued to lead to substantially lower heat- and cold-related mortality projections, since all future demographic scenarios reflect an increase of the pool of vulnerable populations [29, 35]. For example, current demographic projections reveal that the global population aged 65 years and older will substantially increase from 9% to 16% by 2050 [36]. This could potentially result in a much larger temperature-related burden since older adults are particularly more at risk of temperature-related health effects [37]. Additionally, previous studies on cold-related mortality projections generally observed a decrease in cold-related mortality mainly driven by the reduction in cold days [19, 23, 25, 30, 38]. Moreover, no study has disentangled the contribution of population development from the temperature-mortality trends in nationwide projection studies for both heat and cold.

In Switzerland, heat- and cold-related mortality currently accounts for 0.28% and 6.59% of all deaths respectively, particularly affecting the older age groups [29]. Recent projection studies suggest that heat-related deaths will increase up to 2.50% under Representative Concentration Pathway 4.5 (RCP4.5) [19]. Nevertheless, the previous assessments failed to consider the future dynamics of population, especially the impact of progressive ageing. Recent findings indicate that ageing over the past 50 years has resulted in a significant rise in cold- and heat-related mortality in Switzerland [29]. Hence, it becomes crucial to account for socioeconomic scenarios, given the projected increase in the proportion and size of older adults (>65 years) from 16% to 30% by 2060 in Switzerland [37]. Lastly, heat- and cold-related mortality risk has been found to be highly variable across small regions in Switzerland [39]. Thus, the large heterogeneity in risk must be accounted for to obtain robust projections of temperature-mortality impacts.

In this study, we aim to project future heat- and cold-related mortality under various warming scenarios derived based on a set of emissions and population development scenarios in Switzerland. This is the first nationwide analysis using high-resolution climate data under different warming scenarios and detailed vulnerability estimates in terms of age- and district-specific temperature-related mortality. We also aimed to disentangle the contribution of the changes in climate and population development on the overall projected trend.

## 2 Methods

We quantified heat- and cold-related mortality burden for different scenarios of climate change and population development using a two-step approach, as described in Vicedo-Cabrera *et al* (2019). A detailed explanation of the method is provided in Methods S1. In brief, we first derived district-specific temperature-mortality associations through two-stage time series analysis for two age groups (<75 and ⩾75 years) in Switzerland [22, 40]. In the second step, we projected heat- and cold-related mortality impacts in each district under a set of warming scenarios (i.e., 1.5 °C, 2.0 °C and 3.0 °C) using two emission scenarios (representative concentration pathway, RCP) and two population development projections (Shared Socioeconomic Pathways, SSP). Specifically, we used a combination of RCP4.5 and SSP2 (RCP4.5/SSP2), defined as a ‘Middle of the road’ climate scenario characterized by medium fertility and medium mortality of the population. Similarly, we combined RCP8.5 and SSP5 (RCP8.5/SSP5) representing a more pessimistic scenario characterized by a fossil fuel-based development that stresses technological change, rapid development of human capital and high fertility and low mortality [41].

### 2.1 Estimation of the temperature-mortality associations

We derived the temperature-mortality association for each of the 143 districts and age groups using a two-stage time-series approach. In the first stage, we performed separate time-series analyses in each of the districts. For that, we collected daily all-cause mortality between the 1st of January 1990 and the 31st of December 2010 from the Federal Statistical Office (BFS) for each district in Switzerland. Subsequently, we stratified the mortality by age group (<75 and ⩾75 years) based on the methods as done by previous studies [42, 43]. Following the methodology described in de Schrijver *et al*., 2020, we derived daily mean temperature series in each district for the 1990–2010 study period using high resolution 2 km gridded climate dataset (TabsD) provided by MeteoSwiss [44].

We fitted a quasi-Poisson regression model with a natural spline with 8 degrees of freedom per year and an indicator for the day of the week to control for temporal patterns (i.e., seasonality and long-term trends). We estimated the temperature-mortality association using a distributed lag non-linear model [22, 45]. Specifically, we modelled the temperature-mortality association using a natural spline with three internal knots placed at the 10th, 75th and 90th percentile of the district-specific temperature distribution in the exposure-response dimension, as specified in previous studies [22, 46]. We considered up to 21 d of lags to account for the harvesting effect and more delayed effects which were modelled through a natural cubic spline and three internal knots equally spaced on the log scale. We chose these model specifications based on previous studies [19]. We then reduced the bi-dimensional temperature-lagged-mortality association to a one-dimensional overall cumulative exposure-response function [47].

In the second stage, we pooled the overall cumulative exposure-response association by age group and district through a multivariate meta-regression model [40]. We included as fixed effect predictors the age category, district-level mean temperature and temperature range and an indicator for urban or rural, as defined by the BFS, and a random intercept by region (table S2) [48]. Finally, for each district and age group, we derived the best linear unbiased predictions which is an improved temperature-mortality association based on the first-stage exposure-response function and the pooled estimates (figure S1, table S2) [49]. From the curves, we derived the temperature of minimum mortality (MMT) corresponding to the temperature values with the lowest mortality risk. We used this value as reference for the estimation of the relative risks (RRs) at any other temperature value. A more elaborate description of the methodology can be found in the supplementary file (methods S1).

### 2.2 Quantification of heat- and cold-related mortality under scenarios of climate change and population development

We obtained daily mean temperature simulations between 1980 and 2100 from the CH2018 Scenarios [50]. These are the new generation Swiss climate scenarios that were developed by MeteoSwiss to provide policymakers with reliable, up-to-date and comparable evidence on climate change impacts across sectors [50, 51]. CH2018 scenarios include downscaled EUROCORDEX climate simulations (Coupled Model Intercomparison Project—Phase 5 (CMIP5)) on a set of general circulation model (GCM) for RCP4.5 and RCP8.5 (i.e., 25 and 31 simulations, respectively) at a 2 km grid resolution across Switzerland [50]. This unique high-resolution climate data is meant to better represent the local climate and is highly valuable given the complex orography characteristic for Switzerland (i.e., mountainous areas), as opposed to the global GCM with a coarser horizontal resolution. We created district-specific daily-mean temperature time series for 17 GCMs for RCP4.5 and for 22 GCMs for RCP8.5 using the intersecting grid cells for each district (table S1). Additionally, we bias-corrected the simulated temperature series using the observed temperature series following the method specified by Hempel *et al* 2013. Specifically, this method uses a constant offset that corrects for long-term differences between the simulated data and the historical data [52].

To account for changes in the demographic structure of the population, we collected trends in mortality rates and population size between 2010 and 2100 for Switzerland under two SSPs: SSP2 and SSP5. Data regarding population size were available at the national level, by 5 year bands and for the two age groups from IIASA as shown in figure S2 (https://secure.iiasa.ac.at/web-apps/ene/SspDb/) [53], while the mortality rates were the country-specific mortality rates were obtained from Samir and Lutz [41]. We combined the corresponding age-specific mortality rates with the projected population to derive the projected all-cause mortality per 5 years-bands between 2010 and 2100 for Switzerland. Subsequently, we derived daily all-cause deaths by age groups accounting for seasonality by using the share of deaths by day of the year calculated from the observed mortality and calibrated with the observed annual mortality in each district (figure S3).

Subsequently, we combined the temperature series for each GCM and baseline mortality series by age group developed under RCP4.5/SSP2 and RCP8.5/SSP5 with the age- and district-specific temperature-mortality curves to estimate the corresponding heat- and cold-related mortality impacts by warming target, age group and GCM. Consistent with the IPCC Sixth-Assessment Report, we report mortality impacts for the baseline period (1990–2010) and by a set of warming scenarios corresponding to 1.5 °C, 2.0 °C and 3.0 °C warming for RCP4.5/SSP2 and RCP8.5/SSP5. These scenarios correspond to specific 21 year periods which have been categorized using the climate calculator by Taylor *et al* 2012. This method uses linear averaging over the selected model ensemble to generate a mean global mean temperature time series, from which the corresponding exceedance times were derived [54].

We estimated the average annual attributable deaths (AN) and mortality fractions (AF, as AN divided by the total mortality, reported as %), and the corresponding mortality rate attributable to heat and cold (AN/100 000 people) for each warming target, GCM, RCP/SSP trajectory, district and age group. Heat-related mortality was considered as all temperature-related deaths that occur on days with mean temperatures exceeding the MMT, while cold-related mortality is restricted to days with temperatures below the MMT. Uncertainty was measured in terms of 95% empirical confidence interval (CI) derived through Monte Carlo simulations. Specifically, we produced 1000 simulations of the set of coefficients defining the exposure-response functions and derived a set of 1000 simulated impacts for each GCM by combinations of climate scenario, population scenario, district and age group. We then obtained the 95% CI for the ensemble estimates by climate scenario, population scenario and district from the joint distribution of GCM-specific simulated impacts. In this way, the uncertainty estimates account for the variability across models and the imprecision of the association estimates [55].

Finally, we aimed to disentangle the contribution of population development and progressive warming on the projected temperature-mortality impacts. First, we projected the temperature-mortality burden for each warming target using the specific temperature projections but assuming a constant population corresponding to the baseline period (‘climate-only’ scenario). Subsequently, we estimated the contribution of warming as the difference between the main temperature-mortality impacts under a ‘climate-only’ scenario, and the remaining burden then attributed to changes in population development. In a final step, we performed an additional analysis combining RCP4.5 with the SSP5 to assess the contribution of different socioeconomic scenarios (i.e., SSP2 and SSP5).

## 3 Results

**Figure 1:**
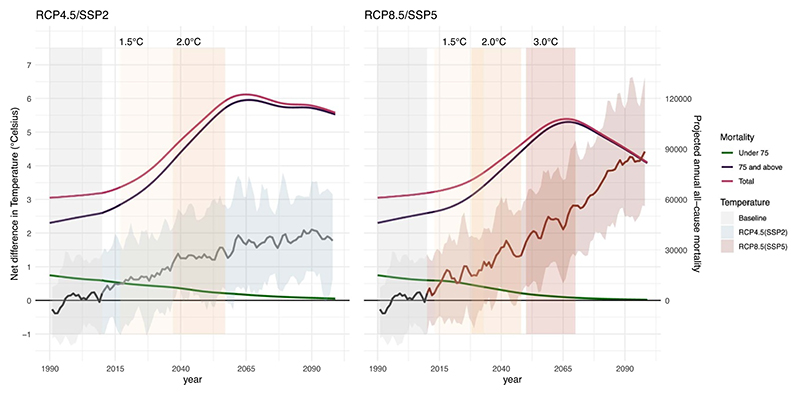
Projected annual mean temperature and age-specific mortality trends by Representative Concentration Pathways (RCP) and Shared-Socioeconomic Pathway scenarios (SSP) and the periods defined for each warming target in Switzerland. The observed temperature for the baseline period is indicated in grey, and the projected annual temperature for RCP4.5/SSP2 is in blue and in red for RCP8.5/SSP5. Age-specific projected annual deaths are represented for under 75 years (green), over and equal 75 years (purple) and total projected annual deaths (pink). The 21 year warming targets are represented with the colored panels (yellow 1.5 °C, orange 2 °C, red 3 °C) and the baseline period in grey.

Table 1 provides a summary of the periods when the warming targets under RCP4.5/SSP2 and RCP8.5/SSP5 are reached, combined with the corresponding projected population development information (i.e., mortality rate, population size and projected all-cause annual deaths). Figure 1 illustrates the warming and projected annual mortality by age group (green <75 years of age, pink ⩾75 years of age, and purple for all age groups). Overall, Switzerland would reach 1.5 °C and 2.0 °C of warming at a later period under a more optimistic scenario of RCP4.5/SSP2 as opposed to RCP8.5/SSP5 (table 1). With RCP4.5/SSP2, a 3.0 °C warming would not be reached before the year 2100, while under RCP8.5/SSP5 this scenario will be possible between 2050 and 2070. Additionally, a substantially larger population is projected under 2.0 °C for RCP8.5/SSP5, than for the same scenario under the RCP4.5/SSP2 (9903 800 vs. 9078 500 respectively), and it is projected to increase up to 11 687 600 in a 3.0 °C warming scenario. Although the population size is projected to be larger under RCP8.5/SSP5, the mortality rate would be substantially lower and decreasing (819 deaths per 100 000 under 2.0 °C of warming) compared to RCP4.5/SSP2 (1101 deaths per 100 000 people). As a result, the annual overall all-cause projected deaths under RCP4.5/SSP2 are projected to be larger with 99 995 all-cause deaths per year under 2.0 °C of warming, compared to 81 103 annual deaths under RCP8.5/SSP5 for the same scenario (figure 1). The age-specific mortality rate, population size and projected annual all-cause mortality per 5 year period between 1980 and 2100 are illustrated in the supplementary file (figure S1).

**Figure 2:**
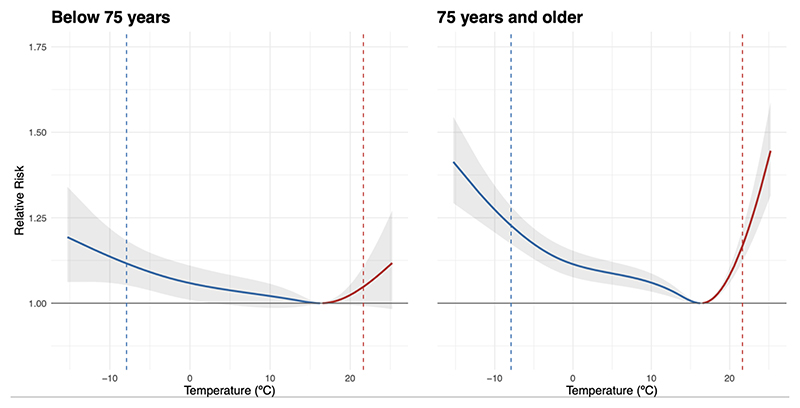
Pooled temperature-mortality association by age group (1990–2010). The pooled temperature mortality association was derived from the meta-analytical model (stage two) for below 75 years, and 75 years and older between 1990 and 2010 in Switzerland, with the vertical line in blue representing the 1st percentile (extreme cold) and in red the 99th percentile (extreme heat).

Figure 2 illustrates the pooled temperature-mortality association over the 1990–2010 period by age group, with the cold-related risks in blue and heat-related risks in red. The population of 75 years and older shows higher vulnerability to extreme cold with a RR of 1.32 (95%CI: 1.24; 1.40) (at 1st percentile of the temperature distribution vs. MMT) compared to the under 75 years group (1.15 (95%CI: 1.06; 1.25). Similarly, the 75 years and older population also show a substantially increased vulnerability for extreme heat (at 99th percentile) with a RR of 1.32 (95%CI: 1.23; 1.42) compared to the group of below 75 years (1.09 (95%CI: 0.99; 1.20)). District-specific temperature-mortality associations have been added to the supplementary materials (figure S1).

Overall, heat-related mortality is projected to increase from 4.3 (95%CI: 1.6; 4.7) annual attributable deaths per 100 000 persons (AD/100 k persons) in the baseline scenario to 13.9 (95%CI: 5.9; 25.0) and 8.9 (95%CI: 3.8; 15.1) AD/100 k persons in Switzerland for RCP4.5/SSP2 and RCP8.5/SSP5 under 2.0 °C, respectively (table S3, figure S4). This will increase up to 16.0 (95%CI: 6.8; 28.1) AD/100 k persons under 3.0 °C of warming. Furthermore, under the 2.0 °C-warming scenario, cold-related mortality is projected to increase from 56.5 (95%CI: 26.4; 83.5) in the baseline scenario to 71.7 (95%CI: 35.2; 104.8) AD/100 k persons under RCP4.5/SSP2 and slightly decrease to 51.2 (95%CI: 24.9; 75.0) AD/100 k persons for RCP8.5/SSP5 and remain constant for 3.0 °C-warming.

**Figure 3:**
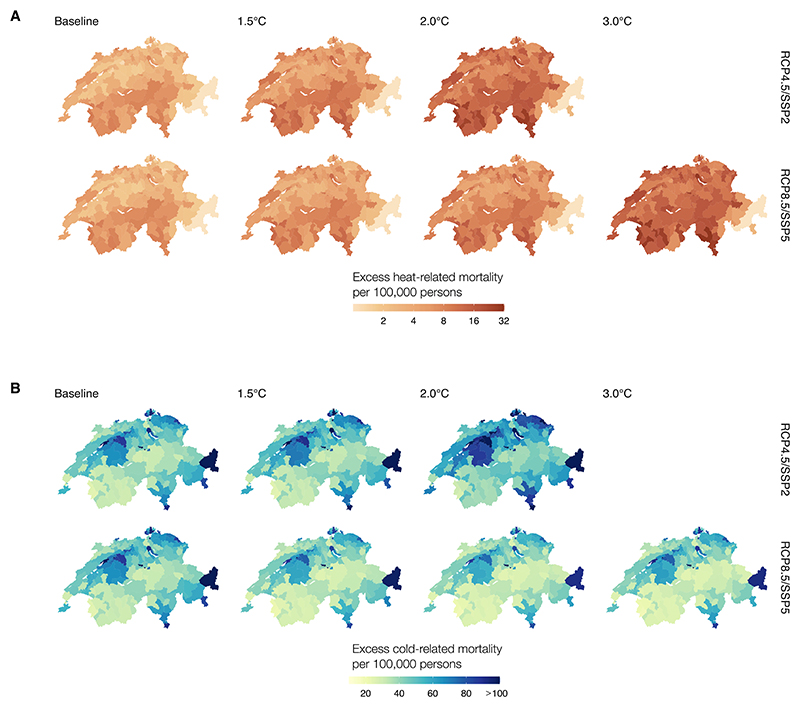
Projected heat- (A) and cold-related (B) excess mortality rates per 100 000 people by warming target for each district in Switzerland for RCP4.5/SSP2 and RCP8.5/SSP5.

Figure 3 illustrates the change in heat- and cold-related mortality rates for each of the 143 districts in Switzerland by warming target for both RCP/SSP compared to the baseline period. Under a 3.0 °C-warming target, heat-related mortality will increase more substantially in Basel-Stadt (32.2 AD/100 k persons), Locarno (31.5 AD/100 k persons) and Zurich (28.4 AD/100 k persons) (table S4), while lowest increases are projected in Davos (5.5 AD/100 k persons) and Visp (6.6 AD/100 k persons). For cold, the largest increases are projected to occur in Solothurn (133.7 AD/100 k persons) and Luzern (107.4 AD/100 k persons) under a 3.0 °C-warming target, while the lowest increases are projected for Monthey (21 AD/100 k persons) and Obwalden (24.5 AD/100 k persons). For heat-related mortality rates under RCP4.5/SSP2, we observe similar patterns to RCP8.5/SSP5, while cold-related mortality will be substantially higher in RCP4.5/SSP2 with the largest impacts in Solothurn (183.4 deaths per 100 000 persons) and Luzern (148.2 deaths per 100 000 persons). District-specific results have been added to the supplementary information (table S4). Finally, we observe larger heterogeneity in cold-related mortality rates between districts as opposed to the heterogeneity between warming targets, while for heat, larger increases between warming targets are estimated, and smaller variation in the heat-related mortality rates between districts for both RCP/SSP scenario (table S4). Besides mortality rates, we observed similar patterns for the corresponding AF (table S5). Under the 2.0 °C-warming target, we observe that cold-related AF is projected to increase from 6.54% (95%CI: 3.05; 9.66) to 8.03% (95%CI: 3.95; 11.75) in RCP4.5/SSP2, while it slightly decreases to 6.21% (95%CI: 3.02; 9.90) for RCP8.5/SSP5. Heat-related AF will increase from 0.50% (0.19; 0.82) to 1.56% (95%CI: 0.66; 2.80) under RCP4.5/SSP2 (2.0 °C) and to 1.80% (95%CI: 0.76; 3.16) RCP8.5/SSP5 (3.0 °C), respectively (table S5, figure S5).

**Figure 4:**
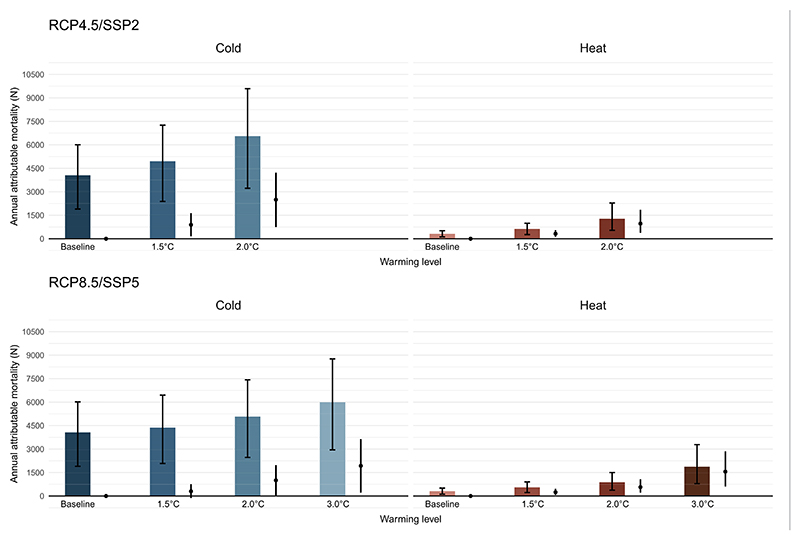
Projected annual heat- and cold-related deaths by RCP/SSP scenario and warming target in Switzerland and for the baseline period (1990–2010), and the corresponding difference in burden from the baseline period (1990–2010). The projected cold-related deaths are represented in blue and in red for heat, with the corresponding 95%CI by warming target. The black line indicates the absolute difference in cold- and heat-related mortality by warming compared to the baseline scenario with the corresponding 95%CI.

Figure 4 illustrates the projected heat- and cold-related annual deaths by warming target derived from each emission and population development scenario (RCP4.5/SSP2 and RCP8.5/SSP5). Under a scenario of 2.0 °C-warming, heat-related mortality is projected to substantially increase from 312 (95%CI:116; 510) to 1274 (95%CI:537; 2284) annual deaths for RCP4.5/SSP2, and to 882 (95%CI: 372; 1499) annual deaths in RCP8.5/SSP5. Under 3.0 °C of warming, the heat-related mortality in RCP8.5/SSP5 is projected to further increase to 1871 (95%CI: 791; 3284) annual heat-related deaths, the equivalent of a sixfold increase compared to the baseline period. When keeping the population constant to the baseline period (i.e., not accounting for population development scenarios), heat-related mortality will still increase but with a lower magnitude, while cold-related mortality will decrease progressively under warmer scenarios (table S6). When accounting for changes in population development, cold-related mortality is likely to substantially increase from 4069 (95%CI: 1898; 6016) to 6558 (95%CI: 3223; 9589) annual deaths for RCP4.5/SSP2 under 2.0 °C of warming compared to the baseline period. Similarly, for RCP8.5/SSP5, cold mortality will increase to 5072 (95%CI: 2466; 7423) annual deaths at 2.0 °C and reach the peak at 3.0 °C of warming with 5997 (95%CI: 2951; 8759) annual deaths, which is a 25% and 47% increase respectively. The annual heat- and cold-related deaths for all warming targets are shown in table S6 and in table S7 the relative increase to the baseline period is represented.

**Figure 5:**
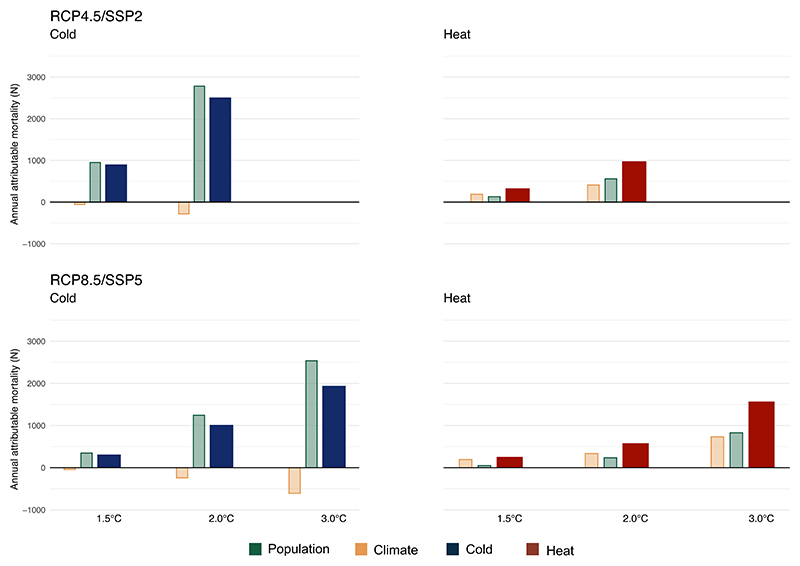
Projected annual heat- and cold-related deaths by RCP/SSP scenario and warming target in Switzerland relative to the baseline (1990–2010), with the contribution of individuals drivers (population development and climate contribution). The contribution of population development is indicated in green, while the contribution of temperature is indicated in yellow. Overall projected heat- and cold-related mortality is indicated in red and blue, respectively.

Figure 5 illustrates the contribution of temperature and the contribution of changes in population development for the projected heat- and cold-related mortality for each combination of RCP/SSP and warming target. We observe that temperature and population development equally contribute to the overall non-optimal temperature-mortality impacts under 2.0 °C of warming, with some variation between RCP/SSP scenarios. For RCP4.5/SSP2, population development resulted in an additional 558 annual deaths (57.8% of the total heat-related deaths), while for RCP8.5/SSP2 this amounts to an additional 235 annual deaths (41.2%) (table S8). For RCP8.5/SSP5, the progressive warming contributes more to the heat-related mortality burden with 335 (58.8%) additional annual deaths under 2.0 °C of warming and increases further to 731 (47.0%) additional annual deaths under 3.0 °C of warming. For cold, in both RCP4.5/SSP2 and RCP8.5/SSP5, the contribution of the warming results in a decrease in the overall projected cold-related mortality with 284 and 240 fewer deaths, respectively, under 2.0 °C of warming, and is projected to further decline with 607 annual cold-related deaths under the 3.0 °C-warming target. In contrast, the contribution of population development is substantially larger than the impact of the progressive warming, resulting in a net increase in overall annual cold-related mortality with a contribution of 2782 (111%) and 1244 (123%) additional annual deaths under 2.0 °C of warming for RCP4.5/SSP2 and RCP8.5/SSP5, respectively. Under 3.0 °C of warming, the contribution of population development is projected to increase to 2536 deaths (131%). Table S8 provides further information on the contribution of each driver for each warming target and RCP/SSP scenario.

Finally, when combining RCP4.5 with SSP5, we estimate that RCP4.5/SSP5 yields a 16.6% lower increase in annual heat-related mortality compared to RCP4.5/SSP2 (tables S9 and S10). Moreover, the discrepancy for annual cold-related mortality between RCP4.5/SSP2 and RCP4.5/SSP5 is larger, where RCP4.5/SSP5 yields a 33.1% lower increase in cold-related mortality compared to RCP4.5/SSP2. The results for the comparison between RCP4.5/SSP2 and RCP4.5/SSP5 are included in the supplementary information (tables S9 and S10).

## 4 Discussion

In this nationwide projection study, we found that both heat- and cold-related mortality will substantially increase under all climate change scenarios in Switzerland. For the same warming level, the increase in burden will depend on the emission and population development scenario. In particular, we observe that RCP4.5/SSP2 will yield larger heat- and cold-related mortality in the short term due to the faster change in population development. However, under a 3.0 °C-warming level, RCP8.5/SSP5 will lead to substantially larger non-optimal temperature-related mortality corresponding to a 6-fold increase for heat and a 50% increase for cold. We observed that changes in population development are the main driver for the increase in cold-related mortality under various warmer scenarios, ultimately outweighing the contribution of the decrease in cold days due to climate change. In contrast, population development and increasing temperatures will both exacerbate the increase in heat-related mortality. Swiss regions will be differently affected, with the districts Zurich, Basel and Ticino showing larger increases in heat-related mortality, and Luzern and Solothurn for cold-related mortality.

Previous projection studies accounting for population development observed similar results for heat-related mortality, with increasing impacts in China [56], the United Kingdom [57], the United States [58] and South Korea [59]. For Switzerland, two previous multi-location studies exploring the temperature-mortality impacts by climate change scenario observed a slightly larger increase in heat impacts compared to our study [19, 60]. For RCP8.5 under 3.0 °C of warming in Switzerland, Vicedo-Cabrera *et al* 2018 observed an additional heat-related mortality fraction of 2.86% (95%CI: 0.96; 5.13) (compared to the baseline), and Gasparrini *et al* 2017 observed an increase to 2.5% (95%CI: 0.8; 5.1), while we estimated an increase to 1.80% (95%CI: 0.76; 3.16). This discrepancy can be explained by the included study locations, where Vicedo-Cabrera *et al*., 2018 and Gasparrini *et al*., 2017, only included the 8 main cities in Switzerland. Another study by Stalhandske *et al* 2022 estimated that by 2050, heat-related mortality would be 1093 annual deaths, which is somewhat lower than our estimates (1871 (95%CI: 791; 3284)) for 3.0 °C of warming that corresponded to the 2050–2070 period [61]. This difference can be explained by two possible factors: first, differently to Stalhandske *et al*., we accounted for population development projections, thus potentially leading to larger heat impacts. Second, we derived detailed estimates of vulnerability to heat and cold in each district, while Stalhandske *et al* derived an average nationwide temperature-mortality association from data on 8 cities, thus, not accounting for the heterogeneity in risks across areas in Switzerland [39, 62]. The effect of differential vulnerability becomes particularly evident in this study, as the spatial variability in heat- and cold-related mortality impacts seem to be larger between districts than over time (i.e., across progressively warmer scenarios), as shown in previous assessments [29, 39].

Previous studies projected a decrease in cold-related mortality under warmer scenarios and argued that this decrease could potentially outweigh the increase in heat-related mortality in cooler regions [19, 23, 24, 30, 60]. In particular for Switzerland, multi-location studies observed a substantial decrease in cold-related mortality, yet, these studies expected an overall increase in non-optimal related mortality [19, 60]. However, these studies did not consider changes in the population under warmer scenarios, thus not accounting for a potential increase in the pool of vulnerable populations. Our results indicate that population development will reverse the reduction in cold-related mortality observed due to the progressive increase in temperature leading to an overall increase in burden under a warming climate. Previous studies that also accounted for population development observed an attenuation or a net increase in overall temperature-mortality impacts, in line with our results [30, 63–65]. In contrast, changes in population development and climate will similarly contribute to the increase in heat-related mortality, with slight differences depending on the RCP/SSP scenario. A recent literature review concluded that not including population development and demographic change could underestimate the projected heat-related mortality by 64% on average, which is in line with our results [66]. Thus, our findings highlight the need to account for changes in population development to provide more realistic estimates of health impacts under future climate change scenarios.

Under the same warming levels, we observed that the increase in heat- and cold-related mortality burden slightly varied depending on the RCP/SSP. In particular, a stronger increase in heat-related mortality was observed under RCP4.5/SSP2 compared to RCP8.5/SSP5 under 2.0 °C of warming. This pattern could be explained by the different projected population structures and thus, different projected annual deaths (i.e., the difference in mortality rate between the two scenarios) between RCP4.5/SSP2 and RCP8.5/SSP5 (figure 1, table 1). Although RCP8.5/SSP5 is projected to have a much larger population, the mortality rate is substantially lower, leading to lower projected annual deaths compared to RCP4.5/SSP2. Therefore, although the level of warming is similar across RCPs/SSP, the number of vulnerable individuals to heat and cold differ, resulting in larger impacts for RCP4.5/SSP2 in the short term due to a higher mortality rate. Moreover, according to the results, when using RCP4.5/SSP5 instead of RCP4.5/SSP2, we observed a somewhat lower increase in heat-related mortality under RCP4.5/SSP5, while the increase in annual cold-related mortality impacts substantially decreased compared to RCP4.5/SSP2, therefore emphasizing once more the considerable role of population development on the projected temperature-mortality impacts.

Overall, this study presents a rigorous evaluation of heat- and cold-related mortality projections under a set of climate change and population development scenarios at a national scale employing unique datasets and state-of-the-art methods in climate epidemiology. Specifically, we used high-resolution downscaled-climate projections data which is key to obtain robust impacts in the complex Swiss orography. Second, leveraging high-resolution temperature-mortality data, we estimated detailed temperature-mortality associations at the district level throughout Switzerland, and thus, obtaining a more accurate representation of the vulnerability of the population. Third, we included changes in mortality rate and population size to project future temperature-related mortality impacts more accurately. Conversely, the impact estimates did not account for changes in vulnerability to heat and cold (i.e., adaptation) in future decades. This can be considered a string and potentially unrealistic assumption since there is evidence showing attenuation of risk across time in retrospective studies in Switzerland [29]. The implementation of heat-adaptation measures in the coming decades might result in a further reduction in heat mortality risk [67, 68]. To date, only a few studies have attempted to include adaptation when projecting future-mortality impacts, although these were largely based on strong and potentially arbitrary modelling assumptions [58, 69, 70]. However, recently, new modelling approaches have been proposed to derive adaptation scenarios based on projections of vulnerability factors following specific storylines of socio-economic development [26]. A further constraint within this research pertains to the utilization of high-resolution CMIP5 climate simulations rather than the more recent CMIP6 simulations. We believe, though, the coarse resolution of the available CMIP6 global simulations would not be suitable given to the complex Swiss terrain leading to a highly variable climate at a fine scale. Recent investigations comparing CMIP5 and the CMIP6 data have indicated that the former tends to somewhat underestimate the magnitude of warming for every climate scenario in Central Europe [10]. Thus, we acknowledge that, if this pattern holds for the Switzerland, our projected health impacts might be conservative. Lastly, it is important to note that our study relied solely on two age categories (<75 and ⩾75 years) to model future impacts, primarily due to limited statistical power. Despite this limitation, we believe that our approach adequately captures the range of vulnerability across age groups and temporal shifts in the population’s demographic structure, as the predominant effects of temperature are observed among older adults [42, 43, 71].

## 5 Conclusion

In this nationwide study, we found that both heat- and cold-related mortality will substantially increase under all climate change scenarios in Switzerland. Moreover, our findings suggest that changes in population development will reverse the reduction in cold-related mortality due to warmer temperatures. Additionally, the combination of changes in population development and progressive warming will lead to a substantially larger heat-related mortality burden. Thus, our findings suggest that substantial societal adaptation measures are urgently needed to counteract the impact of population changes and progressively warmer temperatures, and thus, reduce the impact of climate change on health in Switzerland.

## Supplementary Material

Supplementary material

## Figures and Tables

**Table 1:** Summary description of the 21 year periods when specific warming targets are reached for each RCP/SSP, and the corresponding annual projected population, annual mortality rate and average number of deaths per year in Switzerland. The mortality rate is expressed as the number of annual all-cause deaths per 100 000 people.

RCP/SSP	Warming level	Period	Population	Mortality rate	Projected annual deaths
Baseline	—	1990/2010	7204 055	868	62 551
RCP4.5/SSP2	1.5 C	2017–2037	8307 600	936	77 752
	2.0 C	2037–2057	9078 500	1101	99 995
RCP8.5/SSP5	1.5 C	2013–2033	8471 500	851	72 701
	2.0 C	2028–2048	9903 800	819	81 103
	3.0 C	2050–2070	11 687 600	795	92 868

## Data Availability

The data that support the findings of this study are openly available to partially replicate the analysis at the following URL/DOI: https://doi.org/10.48620/355.

## Code availability

R Code is publicly available on GitHub of the first author of the publication to partially replicate the analysis (https://github.com/evandeschrijver/PhD__open/tree/main/ERL_tempmort_projections_CH_2023).
