# Supplementary material for: Nationwide projections of heat- and cold-related mortality impacts under various climate change and population development scenarios in Switzerland
Source: Environ Res Lett. Author manuscript; Available in PMC 2024 Jun 7. (PMC7616072; doi:10.1088/1748-9326/ace7e1)
Supplement: Supplementary material [file EMS194684-supplement-Supplementary_material.pdf]

Nationwide projections of heat- and cold-related mortality impacts under various climate change and population development scenarios in Switzerland

Evan de Schrijver[1-3], Sidharth Sivaraj[1,2], Christoph C. Raible[2,4], Oscar H. Franco[1,5], Kai Chen[6,7] , Ana M. Vicedo-Cabrera[1,2]

1. Institute of Social and Preventive Medicine (ISPM), University of Bern, Bern, Switzerland
2. Oeschger Center for Climate Change Research (OCCR), University of Bern, Bern, Switzerland.
3. Graduate School of Health Sciences (GHS), University of Bern, Bern, Switzerland
4. Climate and Environmental Physics, Physics Institute, University of Bern, Bern, Switzerland
5. Julius Center for Health Sciences and Primary Care, University of Utrecht Medical Center, Utrecht, The Netherlands
6. Department of Environmental Health Sciences, Yale School of Public Health, New Haven, Connecticut, United States
7. Yale Center on Climate Change and Health, Yale School of Public Health, New Haven, Connecticut, United States

Methods S1.

First stage:
The aim in the first stage is to estimate the associations between temperature and mortality specific to each location, for this, we conducted separate time-series analyses using generalized linear models. We utilized observed temperature and mortality data between 1990-2010, also considered the baseline period.

In this analysis, we employed a quasi-Poisson regression approach with a quasi-likelihood that scaled the standard deviation of the coefficients in proportion to the potential overdispersion.

Generally, we can model the temperature-mortality association through a quasi-Poisson regression which takes the following form:

$$g\left[\mathrm{E}\left(\mu_t\right)\right]=\alpha+f\left(x_t, \ell ; \theta\right)+\sum_{j=1}^J s_j\left(t ; \gamma_j\right)+\sum_{p=1}^P h_p\left(z_{pt} ; \eta_p\right)$$

Where g illustrates the log link function of the expected outcome, $f(x_t, \theta)$ is the approximate integral of the temperature-mortality association with the exposure of interest x at time t accounting for the lagged effects l (up to 21 days of lag). s_j is the smoothing function capturing the effect of confounding variables that slowly change over time, which can be characterised as seasonality or changes in long term trends, defined by parameter β_j and finally h_p representing general confounding variables. To address seasonality, we employed a natural spline with 8 degrees of freedom (d.f.) per year. Furthermore, an indicator for the day of the week was included.

To capture the complex nonlinear and lagged dependencies often observed in temperature-mortality studies, we employed distributed-lag nonlinear models (DLNMs). DLNMs allow for the inclusion of delayed effects of time-varying exposures and enable the quantification of net effects over a predefined lag period.

Following the DLNM methodology, we modeled the bidimensional exposure-lag-response association by combining two functions within a so-called cross-basis term. Specifically, we used a natural spline function with three internal knots at the 10, 75th and 90th of the location-specific temperature distribution to model the exposure-response curve. For the lag-response dimension, we utilized a natural spline function with three internal knots spaced equally in the logarithmic scale over a 21-day lag period.

Moreover, to model the delayed effects of temperature on mortality we follow the notation from Gasparrini et al., 2010, which takes the following form:

$$f\left(x_t, \ell ; \theta\right)=\int_{\ell=\ell_0}^L g \cdot w\left(x_{t-\ell}, \ell ; \theta\right) \simeq \sum_{\ell=\ell_0}^L g \cdot w\left(x_{t-\ell}, \ell ; \theta\right)$$

Where the function $g \cdot w(x_{t-1}, l)$ combines the exposure $g(x)$ and the corresponding lag-response function $w(l)$ of the DLNM. Thus, it represents the cumulative risk over the lag period.

The resulting bidimensional set of coefficients from each location was then consolidated across the lag dimension to derive an overall cumulative exposure-response curve representing the association between heat and mortality over a 21-day lag period.

2nd-stage

The two-stage time series analysis can be divided into several parts: in the *first stage*, the parameters describing the bi-dimensional exposure-lag-response association are summarised into a single parameter estimate while controlling for individual/local covariates, after which in the *second stage* the estimates are combined across different locations into one overall exposure-response function (Sera et al. 2019b). Besides pooling the effects, another strength of the second stage meta-analysis is that Best Linear Unbiased Prediction (BLUP)s allow to explore the association between location specific meta-predictors of the exposure-response association and additionally allows to repredict an enhanced exposure-response function for each location by different sub-groups (Gasparrini et al. 2010). Following the notation of Gasparrini et al. 2011 and Sera et al. 2019b, a general overall pooled exposure-response function model for the multivariate meta-analysis across different cantons or cities can be modelled by using the location-specific associations for each $i = 1, \ldots, m$ location as follows:

$$\hat{\boldsymbol{\theta}}_i \sim \mathrm{N}_k\left(\boldsymbol{\theta}, \mathbf{S}_i + \boldsymbol{\Psi}\right)$$

with S_i representing the within group errors and Ψ_i representing the between location variance-covariance matrices. The k estimated parameters $\hat{\theta}_i$ of the B-spline are used to model the overall-cumulative exposure-response asso- ciation. The in between-location component, θ_i is assumed to be sampled from $N_k(\theta, \Psi)$, where Ψ represents the unknown between-study (co)variance matrix (Gasparrini 2011).

In addition to the marginal level models which pool the overall- cumulative exposure response function, improved location-specific exposure-response functions can be predicted ith more statistical certainty, having borrowed information from on the overall pooled cumulative exposure-response function. Having a repredicted enhanced exposure-response function for each location-specific temperature mortality association based on the overall-cumulative exposure response function, can assist in estimation of the temperature-mortality impacts across locations with little power and large uncertainty. A BLUP can be interpreted as as trade-off between θ_i and $\hat{\theta}_i$, with these estimates of effect sizes borrowing information from various locations. Hence, BLUPs are conditional expectations, given the random-effect multivariate distribution, for which we can write a formula for the BLUP with the predicted $\theta_{b(i)}$ and variance-covariancematrix V $\hat{\theta}_{b(i)}$ in the following way:

$$\hat{\boldsymbol{\theta}}_{b(i)} = \mathbf{X}_i\hat{\boldsymbol{\beta}} + \hat{\boldsymbol{\Psi}}\hat{\boldsymbol{\Sigma}}_i^{-1}\left(\hat{\boldsymbol{\theta}}_i - \mathbf{X}_i\hat{\boldsymbol{\beta}}\right)$$

The BLUP equation represents a trade-off between the fixed effects estimate (Xiβ̂), which represents the overall trend in the data, and the random effects estimate (Ψ̂Σ̂_i^-1(θ̂_i - Xiβ̂)), which represents the deviation of the ith unit from the overall trend where θ̂_b(i) is the BLUP estimate of the parameter for the *i*th unit, Xi is a vector of explanatory variables for the ith unit and β̂ is the estimated regression coefficient vector for the explanatory variables and Ψ̂ is the estimated random effects covariance matrix (Sera et al., 2019). To assess the presence of heterogeneity, we employed multilevel extensions of the Cochran Q test and I^2 statistic. The location-specific associations defined by the BLUPs were utilized in quantifying the impacts of heat-related mortality. All analyses were performed using the R software environment (v.3.5.2) with the dlnm and mixmeta packages.

3rd stage Impact assessment:

Thus far, the analysis of the effect estimate has been based on a measure of association, namely the RR. Although the RR gives a rough estimate how a certain exposure is associated with the baseline, or the reference point, it does not provide the full picture when it comes to the absolute burden of disease onto society, and thus the actual impact since it does not account for the prevalence of the disease nor the exposure. A solution to this is by introducing relative excess measures such as AF and absolute excess measures AN, which are measures of population impact. The AF and AN, combine the relative risk, and more importantly, combine the risk with the prevalence of the exposure to estimate the overall health impacts of an exposure. Thus, it computes the impact and compares this as a proportion to the impact that would not have occurred in the absence of the exposure, also the baseline (Gasparrini et al. 2014). To get an overall idea of the impacts separated by cold and heat, following the notation from Vicedo-Cabrera et al., 2019 we can rewrite the AF for heat and cold-related mortality in the following way:

$$\mathrm{AF}^{-}_{x,t} = 1 - \exp\left(- \sum_{\ell=\ell_0}^{L} I\left(x_{t-\ell} < x_0\right) \beta_{x_{t-\ell},\ell} \right)$$

$$\mathrm{AF}^{+}_{x,t} = 1 - \exp\left(- \sum_{\ell=\ell_0}^{L} I\left(x_{t-\ell} > x_0\right) \beta_{x_{t-\ell},\ell} \right)$$

where x_{t-1} temperature represent the temperature for each modelled GCM series at intensity x, taking into account the lagged effects, x_0 is the temperature of minimum mortality and $AF^{-}{}_{x,t}$ and $AF^{+}{}_{x,t}$ represent the separated excess cold-related mortality fraction and excess heat-related mortality fraction respectively, and $\beta x_{t-\ell,\ \ell}$ represents the the DLNM.

Table S1. CMIP5 General Circulation models derived from CH2018 at a downscaled resoltuon of 2km.

RCP	Model Name	Institution
RCP4.5	CLMCOM-CCLM4	ECEARTH, HADGEM, MPIESM
	DMI-HIRHAM	ECEARTH
	KNMI-RACMO	ECEARTH, HADGEM
	MPICSC-REMO	MPIESM
	MPICSC-REMO2	MPIESM
	SMHI-RCA	CCCMA, CSIRO, ECEARTH, GFDL, HADGEM, IPSL, MIROC, MPIESM , NORSEM
RCP8.5	CLMCOM-CCLM4	ECEARTH, HADGEM, MPIESM
	DMI-HIRHAM	ECEARTH
	KNMI-RACMO	ECEARTH, HADGEM
	MPICSC-REMO	MPIESM
	MPICSC-REMO2	MPIESM
	SMHI-RCA	CCCMA, CSIRO, ECEARTH, GFDL, HADGEM, IPSL, MIROC, MPIESM , NORSEM
	ICTP-REGCM	HADGEM
	CLMCOM-CCLM5	ECEARTH, HADGEM, MPIESM, MIROC

Table S2. Second-stage meta-regression model. The significance test for the predictors was derived using the Wald-test (p-value), the multivariate Cochran Q-test for heterogeneity (p-value), and I^2 statistic (%) in the different multivariate meta-regression models. The model selection was in part based on the Akaike Information Criteria (AIC) while also taking the Wald-test, I^2 and Q-test into consideration.

**Model**	**AIC**	**I^2**	**Cochran Q-test**	**Age**	**Tmean**	**Range temp**	**Urban/rural**	**Random effects**
**Crude model (no variables)**	2144	3.0%	0.22					
**+ age**	2137	1.9%	0.32	<0.01				
**+ age + average temperature**	2128	1.1%	0.39	<0.01	0.01			
**+ age + average temperature + temperature range**	2131	1.2%	0.39	<0.01	0.18	0.57		
**+ age + average temperature + temperature range + urban/rural**	2131	1.0%	0.40	<0.01	0.22	0.55	0.22	
**+ age + average temperature + temperature range + urban/rural + random effects (region)**	2131	1.2%	0.39	<0.01	0.22	0.51	0.42	Incl.
**+ age + average temperature + temperature range + urban/rural + random effects (Canton)**	2134	1.0%	0.40	<0.01	0.20	0.54	0.33	Incl.

Table S3. Projected excess heat- and cold-related mortality rate per 100,000 people by RCP/SSP scenario for temperature projections combined with population development projections by warming level in Switzerland compared to the 1990-2010 period.

RCP	Scenario	Scenario	Heat	Cold
**RCP4.5/SSP2**	Inc pop. development	Baseline	4.3 (1.6; 7.1)	56.5 (26.4; 83.5)
		Scen1.5C	7.5 (3.1; 11.7)	58.9 (28.3; 86.3)
		Scen2.0C	13.9 (5.9; 25.0)	71.7 (35.2; 104.8)
**RCP8.5/SSP5**	Inc pop. development	Baseline	4.3 (1.6; 7.1)	56.5 (26.4; 83.5)
		Scen1.5C	6.4 (2.6; 10.4)	50.4 (24.0; 74.4)
		Scen2.0C	8.9 (3.8; 15.1)	51.2 (24.9; 75.0)
		Scen3.0C	16.0 (6.8; 28.1)	51.3 (25.3; 75.0)

Table S4. Projected excess heat- and cold-related mortality rate per 100,000 people by RCP/SSP for each District for temperature projections combined with population development projections by warming level in Switzerland compared to the 1990-2010 period.

**RCP4.5/SSP2**	**Heat**			**Cold**		
**District**	**Baseline**	**1.5**	**2.0**	**Baseline**	**1.5**	**2.0**
Appenzell Innerrhoden	2.6	4.6	8.5	37.5	39.2	46.7
Basel-Stadt	9.1	15.6	28	75.2	81.3	99
Genève	4.2	7.5	14	55.1	58.2	69.5
Glarus	5.6	8.3	13.9	36.8	37.8	44.3
Neuchâtel	5.7	9.9	17.6	49.4	52.8	63.7
Nidwalden	4	6.9	12.8	32.6	34.4	41.3
Obwalden	3.6	5.7	10.2	29.4	30.4	35.8
Uri	8.1	11.2	17.2	32	33	38.7
Zug	2.7	5	9.7	51.5	54.4	65.6
Surselva	6.4	8.9	13.5	37.1	38.4	45.1
Interlaken-Oberhasli	7.3	9.4	13.5	40.5	41.9	49.2
Engiadina Bassa/Val Müstair	-0.3	-0.9	-2.1	112.1	112.5	130.8
Maloja	-0.3	-0.8	-1.8	58.9	56.8	64
Bern-Mittelland	3.5	6.5	12.5	66.3	71.2	86.6
Prättigau/Davos	1.7	3	5	42.8	39	42.1
Visp	3.9	5	7	30	31.6	37.6
Frutigen-Niedersimmental	6.5	8.9	13.8	36.3	37.3	43.6
Jura-Nord vaudois	3.7	6.6	12.3	46.3	48.8	58.1
Emmental	1.8	3.5	6.9	67.7	69.5	81.9
Albula	1.9	3	4.5	54.1	52.1	58.4
Entremont	7	9.7	14.9	32.2	33.1	38.8
Viamala	2	3.4	5.7	52.4	48.5	52.7

Locarno	8.4	15	27.6	61.2	65.4	78.7
Goms	8.1	10.6	15.5	40.8	43	51.1
Obersimmental-Saanen	5.7	8.1	12.6	32.5	33.8	39.6
Vallemaggia	5.8	9.1	16	33.7	34.8	40.7
Jura bernois	1.9	3.7	7	51.8	52.7	61.8
Sarganserland	2.2	4.3	8.5	38.7	37	41.2
Schwyz	3	4.8	8.5	31.7	32.7	38.5
La Gruyère	4	6.3	10.8	32.2	32.9	38.4
Moesa	2.4	3.3	5.5	48.9	51.4	60.8
Toggenburg	2.1	4.2	8.1	48.4	49.8	58.8
Leventina	7	10.4	17.5	43.8	45.5	53.6
Aigle	5.9	8.7	14.5	28.8	29	33.2
Brig	6.6	10.8	19	34.7	36.9	44.1
Entlebuch	3.3	5.7	10.3	36.1	37.7	44.8
Sierre	8.2	13.4	23.2	32.8	34.2	40.6
Hérens	8.6	11.8	17.8	31.9	32.5	37.9
Morges	3.2	6	11.4	47	50	60.1
Blenio	7.6	11.2	18.5	48.8	51.2	60.7
Lugano	7.7	13.1	23.7	68.5	72.4	87
Willisau	1.9	3.7	7.4	51.6	54.7	65.9
Seeland	1.9	3.8	7.4	69.4	71.6	84.9
Leuk	4.5	6.9	11.5	30.1	31.4	37.3
Porrentruy	4.7	7.7	13.6	39.7	41.7	49.9
Oberaargau	1.8	3.6	7.2	87	90	106.8
Thun	4.9	9	17.1	63.9	68.8	83.6
Nyon	3.3	5.7	10.3	29.4	30.9	36.8
Delémont	3.3	5.8	10.6	36.1	38.6	46.7
Sursee	1.7	3.2	6.4	43.2	45.5	54.7
Plessur	5.8	10.1	18.3	39.5	41	48.6
Riviera-Pays-d'Enhaut	8.5	14	24.5	42.3	44.8	53.4
Frauenfeld	1.6	3	6.1	65.5	67.2	79.6
Raron	4.2	5.9	9.5	32	32.7	38.3
Monthey	9.2	14.7	25.4	24.5	25.5	30.2
Sense	1.8	3.4	6.6	34.5	35.9	42.8
Martigny	7.3	13.3	24.9	38.1	40	47.6
See-Gaster	2.2	4.3	8.4	47.7	49.5	59
Winterthur	4.8	8.6	16.1	62.7	67.1	81.4
Bernina	0.9	1.5	2.5	82.7	79.1	87.6
Conthey	4.5	7.7	14	29.8	30.7	36.1
Gros-de-Vaud	1.9	3.6	6.9	33.9	35.8	42.9
Weinfelden	1.5	3	6	68.1	70.1	83.1
Bellinzona	5.4	9.8	18.4	62.1	66.1	79.5
Luzern-Land	3.2	5.9	11.2	45.4	48.3	58.3
La Sarine	3.3	6	11.3	52.4	55.2	66.2
Werdenberg	2.4	4.9	9.7	44.4	46.5	55.5
Imboden	3.9	7.3	13.9	31.7	32.8	38.9

Les Franches-Montagnes	2.7	4.4	7.7	34.3	35.6	42.3
Broye-Vully	2.1	4.2	8.3	59.2	61.5	73.1
Saint-Maurice	6.7	10.5	18.2	24.2	24.5	28.2
March	3.1	5.5	10.1	36.9	38.9	46.8
Bülach	3.2	5.6	10.4	43.6	45.7	54.7
Hochdorf	1.7	3.3	6.7	52.6	55.2	66.3
Hinwil	3.5	6.3	11.8	50	52.8	63.7
Landquart	2.7	5.6	11.1	37.7	39.7	47.6
La Glâne	2	3.9	7.6	47	49.1	58.5
Andelfingen	2.7	5.2	10.1	61.6	65.6	79.4
Pfäffikon	2.9	5.1	9.5	38.2	40.1	48.2
See	2.1	4	7.9	55.1	57.8	69.3
St. Gallen	4.5	8.2	15.3	62	66.2	80.4
Laufenburg	2.9	5.4	10.1	43	45.4	54.6
Baden	3.5	6.2	11.7	50.2	53.1	64.1
Dielsdorf	2.3	4.1	7.6	32.4	33.4	39.7
Riviera	3.2	5.2	9.4	38.8	40.2	47.2
Brugg	2.5	4.6	8.8	48.1	51.2	62
Wil	3.2	5.8	11	55.9	59.2	71.5
Zofingen	3.2	6	11.5	63.5	67.3	81.2
Sissach	2.6	4.8	9.3	41.1	43.6	52.5
Lavaux-Oron	6	10.7	19.8	53.9	58.3	70.7
Thal	1.7	3.2	6.3	59	59.8	70
Muri	2.2	4	7.7	43.9	45.7	54.7
Rheintal	4.6	8.4	15.7	61.1	63.8	76.3
Münchwilen	1.2	2.5	5	54.9	55.3	64.6
Hinterland	5	9	16.6	52.3	56.1	68.2
La Veveyse	3.1	5.1	9.1	29.5	30.2	35.5
Sion	6.3	11.7	22.1	39.9	42.6	51.2
Zurzach	2.4	4.5	8.8	53.8	56.4	67.6
Kreuzlingen	1.6	3.1	6.2	71.8	73.4	86.8
La Broye	2.3	4.4	8.7	58.4	60.3	71.5
Uster	3.3	5.8	10.7	42.3	44.2	53
Bremgarten	3.6	6.3	11.7	47.7	49.9	59.8
Lebern	3.2	5.9	11.2	71.2	75.8	91.7
Affoltern	2.9	5.1	9.5	38	39.7	47.5
Rheinfelden	5.6	9.5	17.1	48.6	51.4	61.8
Einsiedeln	3.8	6.8	12.5	49.2	52.6	63.6
Waldenburg	2.3	4.3	8.4	39.8	42.2	51
Aarau	5.4	9.6	17.7	65.1	70	85.2
Horgen	4.5	8.2	15.5	61.6	65.9	80
Lenzburg	3	5.5	10.5	55.8	58.7	70.6
Mendrisio	7.1	12.3	23.1	91.4	97.7	118.3
Kulm	3.6	6.6	12.7	59.8	63.5	76.8
Biel/Bienne	5	9.2	17.2	97.9	104.1	125.7
Arlesheim	5	8.7	15.6	45.7	49.2	59.9

Schaffhausen	4.3	8.1	15.5	94.7	100.7	122
Zürich	7.4	13.3	24.9	92.6	99.8	121.8
Arbon	4	7.2	13.6	84.3	89.6	108.6
Thierstein	2.1	4.1	7.8	48.8	50.6	60.3
Liestal	4.4	7.6	13.8	46.1	49.2	59.5
Meilen	5.7	10.5	19.8	74.7	80.4	98
Laufen	4.2	7.2	12.9	38.5	41.1	49.8
Olten	3.6	6.7	12.7	78.9	84.2	102.1
Wasseramt	3.1	5.9	11.1	85	89.8	108.1
Gösgen	2.7	5	9.6	50.5	53.7	64.9
Lausanne	6	10.5	19.4	52.1	55.9	67.6
Bucheggberg	1.3	2.7	5.4	74.5	77.3	92.1
Gäu	1.2	2.4	4.7	65.7	65.4	75.9
Mittelland	2.7	5.3	10.2	44.9	48.2	58.6
Dietikon	4.3	7.6	14.3	54.6	57.7	69.5
Rorschach	4.6	8.4	15.8	66.7	71	86
Dorneck	3.5	6.1	11.1	40.4	42.4	50.9
Unterklettgau	2.5	4.9	9.7	85.7	90.6	109
Vorderland	3.1	6.1	11.9	57.3	61.4	74.4
Höfe	2.7	5	9.5	51.4	54.3	65.4
Schleitheim	1.5	2.9	5.8	58.5	59.9	70.6
Reiat	2.4	4.4	8.4	53.9	56.9	68.7
Küssnacht (SZ)	3	5.7	10.8	49.4	52.6	63.6
Oberklettgau	1.9	3.7	7.4	72.3	75.2	89.7
Stein	2.4	4.8	9.7	82.9	88	106
Luzern-Stadt	6.1	11.5	22.1	112.7	121.6	148.2
Ouest lausannois	5.3	9	16.3	48.7	51.4	61.6
Gersau	3.3	6.6	13.4	76	79.1	94
Solothurn	5.5	10.3	19.9	138.8	150.2	183.4

**RCP8.5/SSP5**	**Heat**				**Cold**			
District	**Baseline**	**1.5**	**2.0**	**3.0**	**Baseline**	**1.5**	**2.0**	**3.0**
Appenzell Innerrhoden	2.6	3.9	5.2	9.9	37.5	33.2	32	32.6
Basel-Stadt	9.3	13.6	17.4	32.2	75.3	68.1	67.4	71.9
Genève	4.2	6.4	8.6	16.6	55.2	49.2	47.8	50
Glarus	5.6	7.1	8.9	15.4	36.8	32.2	30.5	30.1
Neuchâtel	5.9	8.4	10.9	20.3	49.5	44.5	43.5	45.5
Nidwalden	4	5.8	7.8	14.7	32.7	29.1	28.2	29
Obwalden	3.6	4.9	6.3	11.5	29.5	25.8	24.6	24.5
Uri	8.1	9.5	11.3	17.7	32.1	28.1	26.4	26
Zug	2.7	4.3	5.9	11.3	51.6	46.1	44.8	47
Surselva	6.5	7.5	8.9	13.9	37.2	32.7	30.8	30.4
Interlaken-Oberhasli	7.3	8	9	13.1	40.6	35.6	33.5	33.2
Engiadina Bassa/Val Müstair	-0.3	-0.7	-1.2	-3.3	112.5	97.7	90.9	92.2
Maloja	-0.3	-0.7	-1.1	-2.6	59.1	50.1	45	44

Bern-Mittelland	3.5	5.5	7.5	14.7	66.4	60	59	62.4
Prättigau/Davos	1.7	2.4	3.2	5.5	42.9	34.9	30.1	27.3
Visp	4	4.2	4.7	6.6	30.1	26.9	25.4	25.8
Frutigen-Niedersimmental	6.5	7.6	9	14.4	36.4	31.8	29.9	29.4
Jura-Nord vaudois	3.8	5.6	7.5	14.4	46.4	41.1	39.8	41.2
Emmental	1.8	2.9	4.1	8.2	67.9	59.5	56.5	57.9
Albula	2	2.4	3	4.3	54.2	45.8	41	39
Entremont	7	8.1	9.7	15.2	32.3	28.3	26.4	26.2
Viamala	2.1	2.7	3.6	6.1	52.6	43.2	37.5	34.4
Locarno	8.5	12.5	17	31.5	61.4	55.1	53.4	55.8
Goms	8.2	9	10.3	15	41	36.5	34.5	35
Obersimmental-Saanen	5.8	6.9	8.2	13.2	32.7	28.7	27.2	26.8
Vallemaggia	5.9	7.7	10.2	18	33.8	29.5	27.6	27.6
Jura bernois	1.9	3.1	4.3	8.4	51.9	45.1	42.7	43.4
Sarganserland	2.2	3.5	5.1	10.1	38.8	32.4	29.1	27.3
Schwyz	3	4	5.2	9.7	31.7	27.8	26.5	26.5
La Gruyère	4.1	5.4	6.8	12.4	32.3	28	26.5	26.4
Moesa	2.4	2.8	3.5	6.1	49	43.5	41.4	41.8
Toggenburg	2.2	3.4	4.9	9.5	48.5	42.5	40.6	41.3
Leventina	7	8.9	11.2	19.3	44	38.8	36.5	36.5
Aigle	6	7.4	9.2	16	28.9	24.8	23	22.2
Brig	6.6	9.1	12	21.7	34.9	31.3	29.9	30.6
Entlebuch	3.4	4.8	6.3	11.9	36.2	31.9	30.7	31.2
Sierre	8.2	11.1	14.6	26	32.9	29.2	27.6	27.9
Hérens	8.6	10	11.8	18.2	32	27.8	25.8	25.4
Morges	3.2	5	6.9	13.6	47.1	42.1	41.1	43
Blenio	7.7	9.6	11.9	19.9	48.9	43.4	41.3	41.7
Lugano	7.8	11	14.6	26.2	68.6	61.3	59.4	62.5
Willisau	1.9	3.1	4.4	8.7	51.7	46.3	45	47.3
Seeland	2	3.2	4.4	8.9	69.5	61.1	58.5	60.7
Leuk	4.5	5.7	7.3	12.7	30.2	26.7	25.4	25.5
Porrentruy	4.8	6.7	8.5	15.8	39.8	35.1	34.2	35.4
Oberaargau	1.8	3	4.2	8.5	87.2	76.9	73.6	76.4
Thun	4.9	7.6	10.4	20	64.1	58	56.9	59.7
Nyon	3.3	4.9	6.4	12.1	29.4	26.1	25.2	26.2
Delémont	3.3	5	6.5	12.3	36.1	32.4	31.8	33.4
Sursee	1.7	2.7	3.8	7.6	43.3	38.6	37.4	39.2
Plessur	5.7	8.3	11.3	21.4	39.6	34.9	33.2	33.3
Riviera-Pays-d'Enhaut	8.5	11.8	15.3	27.6	42.5	37.8	36.5	37.2
Frauenfeld	1.6	2.6	3.6	7.3	65.6	57.5	54.9	56.8
Raron	4.2	5	6.1	10.4	32.1	28	26.1	25.9
Monthey	9.2	12.4	15.8	28.1	24.6	21.6	20.7	21
Sense	1.8	2.8	3.9	7.8	34.6	30.5	29.3	30.2
Martigny	7.3	11	15.2	29.1	38.2	33.9	32.5	33.1
See-Gaster	2.2	3.6	5	9.8	47.8	42.2	40.6	41.7
Winterthur	4.9	7.4	9.8	18.3	62.8	56.6	55.5	59

Bernina	0.9	1.1	1.6	2.8	83.1	69.4	61.6	58.2
Conthey	4.5	6.3	8.6	16.4	29.9	26.1	24.6	24.5
Gros-de-Vaud	1.9	3	4.2	8.3	34	30.2	29.4	30.7
Weinfelden	1.5	2.5	3.5	7	68.3	59.9	57.3	59.4
Bellinzona	5.4	8.1	11.2	21.1	62.2	55.8	54.1	56.7
Luzern-Land	3.3	5	6.8	13	45.5	40.8	39.8	41.8
La Sarine	3.4	5.2	6.9	13.2	52.4	46.7	45.3	47.4
Werdenberg	2.4	4	5.8	11.7	44.5	39.4	38.1	39
Imboden	3.9	6	8.5	16.3	31.7	27.9	26.6	26.8
Les Franches-Montagnes	2.8	3.8	4.8	9	34.3	30.2	29	29.8
Broye-Vully	2.2	3.5	4.9	10	59.3	52.3	50.3	52.2
Saint-Maurice	6.7	8.8	11.3	20.3	24.3	20.9	19.5	18.9
March	3.1	4.6	6.2	11.7	37	33	32	33.1
Bülach	3.2	4.9	6.3	11.8	43.7	38.7	37.5	39.3
Hochdorf	1.7	2.8	4	7.9	52.7	46.9	45.4	47.6
Hinwil	3.5	5.4	7.2	13.6	50.1	44.7	43.5	45.7
Landquart	2.7	4.5	6.6	13.4	37.8	33.7	32.5	33.3
La Glâne	2	3.2	4.5	9.1	47	41.6	40.1	41.6
Andelfingen	2.8	4.5	6	11.7	61.8	55.4	54.2	57.3
Pfäffikon	2.9	4.4	5.8	10.8	38.2	34	33	34.6
See	2.1	3.4	4.7	9.3	55.2	49.1	47.5	49.8
St. Gallen	4.5	6.9	9.3	17.5	62.1	55.9	54.8	57.7
Laufenburg	3	4.6	6.2	11.9	43.1	38.3	37.3	39.2
Baden	3.6	5.4	7.1	13.5	50.2	44.9	43.8	46.3
Dielsdorf	2.4	3.6	4.6	8.6	32.4	28.5	27.3	28.4
Riviera	3.2	4.4	5.8	10.7	38.9	34.1	32.2	32.4
Brugg	2.5	3.9	5.4	10.4	48.2	43.2	42.3	44.8
Wil	3.2	4.9	6.6	12.4	56	50.1	48.9	51.5
Zofingen	3.3	5.1	6.9	13.2	63.6	56.9	55.5	58.6
Sissach	2.6	4.2	5.7	11.1	41.2	36.7	35.8	37.5
Lavaux-Oron	6	9.1	12.2	23.1	54.1	49	48.3	51
Thal	1.7	2.7	3.7	7.5	59.2	51.3	48.5	49.4
Muri	2.2	3.4	4.6	8.9	43.9	38.9	37.5	39.3
Rheintal	4.6	7.1	9.6	17.8	61.1	54.2	52.4	54.4
Münchwilen	1.3	2.1	2.9	5.8	55	47.6	44.8	45.7
Hinterland	5	7.6	10.2	19.1	52.3	47.3	46.4	48.6
La Veveyse	3.1	4.3	5.7	10.6	29.5	25.7	24.4	24.6
Sion	6.2	9.6	13.5	26	40	35.9	34.8	35.8
Zurzach	2.4	3.9	5.3	10.4	53.9	47.8	46.3	48.6
Kreuzlingen	1.6	2.6	3.7	7.3	71.9	62.8	59.8	61.8
La Broye	2.3	3.8	5.2	10.4	58.4	51.3	49.1	50.9
Uster	3.3	5	6.5	12.1	42.3	37.5	36.3	38.1
Bremgarten	3.7	5.4	7.1	13.2	47.7	42.4	40.9	43
Lebern	3.2	5	6.8	13	71.4	64.1	62.6	66.3
Affoltern	2.9	4.4	5.8	10.9	38	33.7	32.5	34
Rheinfelden	5.7	8.4	10.6	19.5	48.6	43.3	42.2	44.5

Einsiedeln	3.8	5.7	7.7	14.4	49.3	44.4	43.4	45.2
Waldenburg	2.3	3.7	5.1	10	39.9	35.5	34.7	36.4
Aarau	5.6	8.3	10.9	20.3	65.2	58.9	58	61.8
Horgen	4.6	7	9.4	17.9	61.7	55.6	54.6	57.8
Lenzburg	3	4.7	6.3	12.3	55.8	49.7	48.3	50.8
Mendrisio	7.2	10.4	14	25.5	91.7	82.7	80.7	85.9
Kulm	3.6	5.6	7.6	14.7	59.9	53.6	52.4	55.3
Biel/Bienne	5.1	7.8	10.4	20	98.1	88	86	91
Arlesheim	5.1	7.6	9.7	18	45.7	41.3	40.8	43.4
Schaffhausen	4.4	7	9.4	18.2	94.9	85.2	83.3	88.2
Zürich	7.5	11.4	15.1	28.4	92.8	84	82.9	88.5
Arbon	4	6.1	8.2	15.5	84.4	75.9	74.2	78.6
Thierstein	2.2	3.5	4.7	9.4	48.9	43	41.4	42.8
Liestal	4.4	6.6	8.6	15.9	46.2	41.4	40.6	42.9
Meilen	5.8	8.9	12	22.7	74.9	67.7	66.8	71
Laufen	4.3	6.3	8	14.6	38.5	34.5	33.8	35.8
Olten	3.7	5.7	7.7	14.8	79.1	71.2	69.6	73.9
Wasseramt	3.2	5	6.7	13	85.1	76.1	74	78.1
Gösgen	2.8	4.4	5.8	11.2	50.6	45.4	44.3	46.8
Lausanne	6	9	11.9	22.6	52.2	47	46.2	48.7
Bucheggberg	1.4	2.3	3.2	6.5	74.7	66	63.3	65.9
Gäu	1.2	2	2.8	5.6	65.8	56.6	52.7	53.6
Mittelland	2.7	4.4	6.2	12.1	45	40.6	39.9	41.6
Dietikon	4.4	6.6	8.6	16.2	54.7	48.8	47.5	50.1
Rorschach	4.7	7.2	9.6	17.9	66.8	60	58.7	61.8
Dorneck	3.6	5.4	6.9	12.8	40.4	35.8	34.7	36.3
Unterklettgau	2.6	4.2	5.9	11.6	86	76.8	74.6	78.7
Vorderland	3.1	5.1	7.2	14	57.4	51.7	50.8	53
Höfe	2.7	4.2	5.8	11.1	51.5	46	44.8	46.9
Schleitheim	1.5	2.5	3.5	7.1	58.6	51.2	48.8	50.3
Reiat	2.5	3.8	5.1	9.9	54	48.2	47	49.6
Küssnacht (SZ)	3.1	4.8	6.6	12.7	49.5	44.4	43.4	45.7
Oberklettgau	1.9	3.2	4.4	8.8	72.6	64.1	61.8	64.3
Stein	2.4	4.2	5.7	11.4	83.2	74.2	72.6	76.5
Luzern-Stadt	6.2	9.7	13.4	25.8	113	102.5	101	107.4
Ouest lausannois	5.3	7.7	10.1	18.9	48.8	43.5	42.2	44.3
Gersau	3.3	5.5	8	15.9	76.2	67.4	64.7	66.7
Solothurn	5.7	8.8	11.8	22.9	139.3	126.6	125	133.7

Table S5. Projected excess heat- and cold-related mortality fractions by RCP/SSP scenario for temperature projections combined with population development projections by warming level in Switzerland.

RCP	Scenario	Scenario	Heat	Cold
**RCP4.5/SSP2**	Inc pop. development	Baseline	0.50 (0.19; 0.82)	6.54 (3.05; 9.66)
		Scen1.5C	0.91 (0.38; 1.43)	7.17 (3.45; 10.51)
		Scen2.0C	1.56 (0.66; 2.80)	8.03 (3.95; 11.75)
**RCP8.5/SSP5**	Inc pop. development	Baseline	0.50 (0.19; 0.82)	6.54 (3.05; 9.66)
		Scen1.5C	0.81 (0.32; 1.31)	6.33 (3.01; 9.34)
		Scen2.0C	1.08 (0.46; 1.84)	6.21 (3.02; 9.09)
		Scen3.0C	1.80 (0.76; 3.16)	5.78 (2.84; 8.44)

Table S6. Projected excess heat- and cold-related mortality impacts by RCP/SSP scenario for temperature projections combined with population development projections by warming level in Switzerland.

RCP	Scenario	Scenario	Heat	Cold
**RCP4.5/SSP2**	Inc pop. development	Baseline	312 (116; 510)	4069 (1898; 6016)
		Scen1.5C	626 (265; 987)	4951 (2384; 7254)
		Scen2.0C	1,274 (537; 2284)	6558 (3223; 9589)
	Excl. pop. development	Baseline	312 (116; 510)	4069 (1898; 6016)
		Scen1.5C	499 (196; 798)	4030 (1,857; 5996)
		Scen2.0C	719 (265; 11309)	3804 (1720; 5700)
**RCP8.5/SSP5**	Inc pop. development	Baseline	312 (116; 510)	4069 (1898; 6016)
		Scen1.5C	556 (223; 901)	4371 (2076; 6446)
		Scen2.0C	882 (372; 1499)	5072 (2466; 7423)
		Scen3.0C	1,871 (791; 3284)	5997 (2951; 8759)
	Excl. pop. development	Baseline	312 (116; 510)	4069 (1898; 6016)
		Scen1.5C	508 (194; 831)	4033 (1873; 5989)
		Scen2.0C	648 (246; 1116)	3839 (1749; 5733)
		Scen3.0C	1,044 (368; 1899)	3472 (1535; 5232)

Table S7. Difference in projected excess heat- and cold-related mortality impacts by RCP/SSP scenario for temperature projections combined with population development projections by warming level in Switzerland.

RCP	Scenario	Scenario	Heat	Cold
**RCP4.5/SSP2**	Inc pop. development	Baseline	0	0
		Scen1.5C	317 (129; 548)	891 (151; 632)
		Scen2.0C	966 (381; 855)	2498 (747; 4,222)
**RCP8.5/SSP5**	Inc pop. development	Baseline	0	0
		Scen1.5C	244 (66;466)	302 (-131; 759)
		Scen2.0C	570 (218; 1,071)	1,003 (44; 1,975)
		Scen3.0C	1,558 (610; 853)	1,928 (218; 3,635)

Table S8. Contribution of population development and temperature scenario compared to the projected overall excess heat- and cold-related mortality impacts by RCP/SSP scenario in Switzerland compared to the 1990-2010 period.

RCP	Contribution	Scenario	Heat	Cold
**RCP4.5/SSP2**	**Total**	Scen1.5C	318	891
		Scen2.0C	966	2498
	**Temperature**	Scen1.5C	188	-57
		Scen2.0C	408	-284
	**Population**	Scen1.5C	129	948
		Scen2.0C	558	2782
**RCP8.5/SSP5**	**Total**	Scen1.5C	244	302
		Scen2.0C	570	1004
		Scen3.0C	1558	1929
	**Temperature**	Scen1.5C	195	-46
		Scen2.0C	335	-240
		Scen3.0C	731	-607
	**Population**	Scen1.5C	50	348
		Scen2.0C	235	1244
		Scen3.0C	827	2536

Table S9. Projected excess heat- and cold-related mortality impacts for RCP4.5 using different combinations of SSPs (SSP2 and SSP5)

RCP	Scenario	Heat	Cold
**RCP4.5/SSP2**	Baseline	312 (116; 510)	4,069 (1,898; 6,016)
	Scen1.5C	626 (265; 987)	4,951 (2,384; 7,254)
	Scen2.0C	1,274 (537; 2,284)	6,558 (3,223; 9,589)
**RCP4.5/SSP5**	Baseline	312 (116; 510)	4,069 (1,898; 6,016)
	Scen1.5C	574 (239; 907)	4,566 (2,177; 6,713)
	Scen2.0C	1,114 (472; 1,993)	5,733 (2,466; 7,423)

Table S10. Relative change in excess heat- and cold-related mortality impacts for RCP4.5 using different combinations of SSPs (SSP2 and SSP5). The difference percent in increase between RCP4.5/SSP2 and RCP4.5/SSP5 is calculated by ((ScenarioSSP5-ScenarioSSP2)/ScenarioSSP2)*100, while the scenario represents the relative increase compared to the Baseline period.

RCP	Scenario	SSP2	SSP5	% difference
**Heat**	Baseline	0	0	-
	Scen1.5C	314	262	-16.6%
	Scen2.0C	962	802	-16.6%
**Cold**	Baseline	0	0	-
	Scen1.5C	887	497	-43.3%
	Scen2.0C	2,489	1,664	-33.1%

Figure S1. Temperature-mortality association for 143 districts in Switzerland amongst the 75 years and older age group

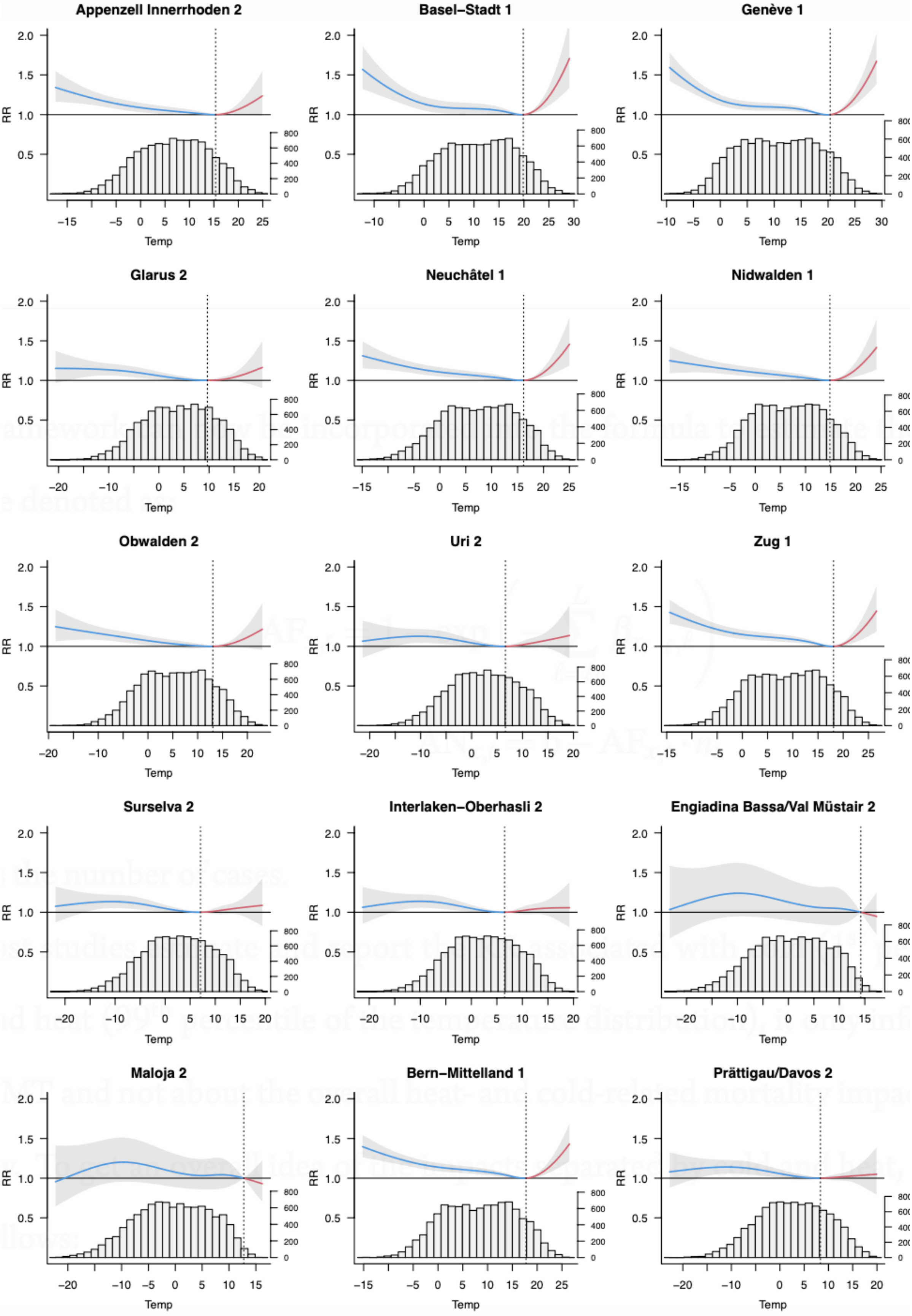

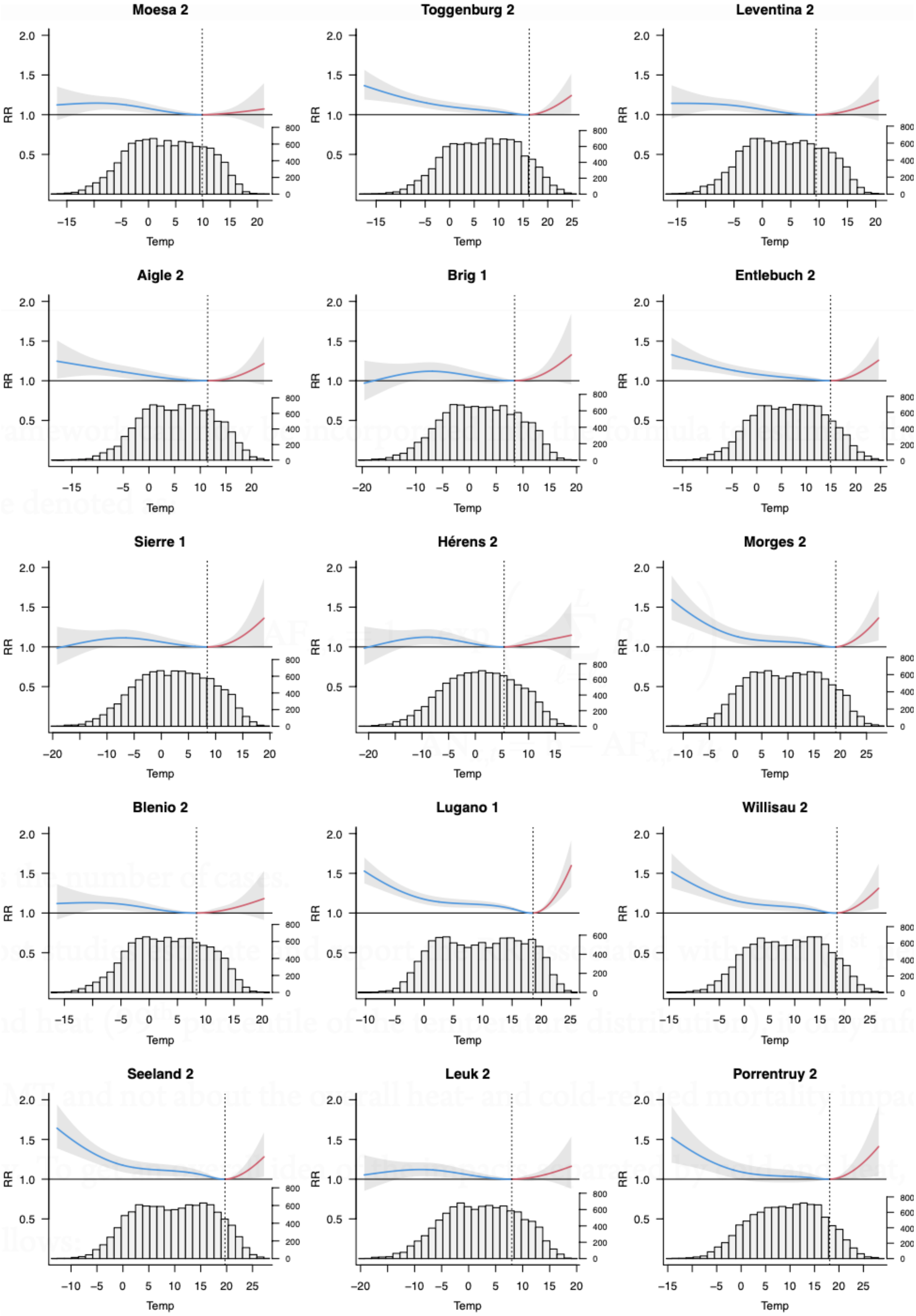
Moesa 2
Toggenburg 2
Leventina 2
Aigle 2
Brig 1
Entlebuch 2
Sierre 1
Hérens 2
Morges 2
Blenio 2
Lugano 1
Willisau 2
Seeland 2
Leuk 2
Porrentruy 2
RR
Temp

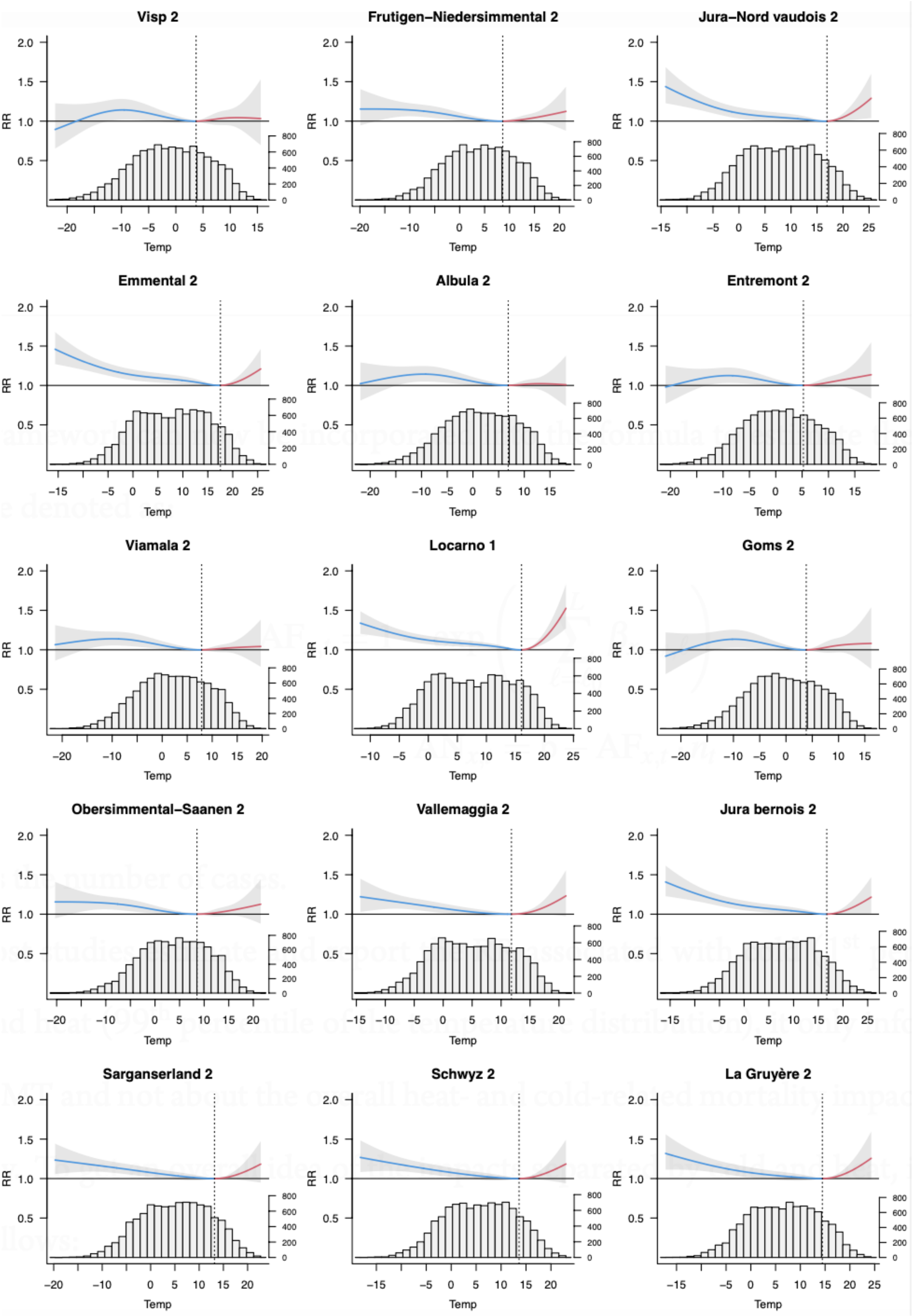
Visp 2
Frutigen–Niedersimmental 2
Jura–Nord vaudois 2
Emmental 2
Albula 2
Entremont 2
Viamala 2
Locarno 1
Goms 2
Obersimmental–Saanen 2
Vallemaggia 2
Jura bernois 2
Sarganserland 2
Schwyz 2
La Gruyère 2
RR
Temp

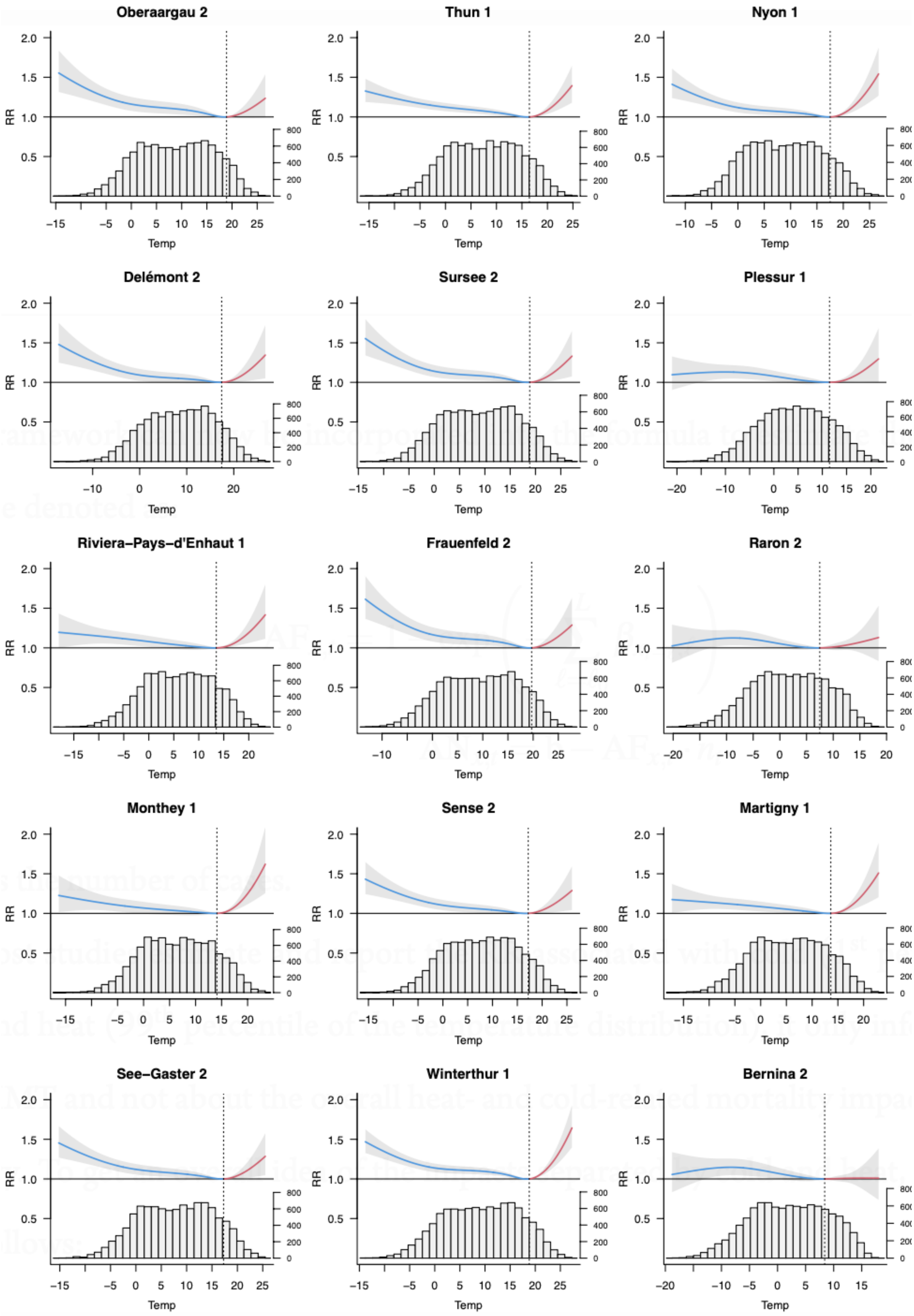
Oberaargau 2
Thun 1
Nyon 1
Delémont 2
Sursee 2
Plessur 1
Riviera–Pays–d'Enhaut 1
Frauenfeld 2
Raron 2
Monthey 1
Sense 2
Martigny 1
See–Gaster 2
Winterthur 1
Bernina 2
RR
Temp

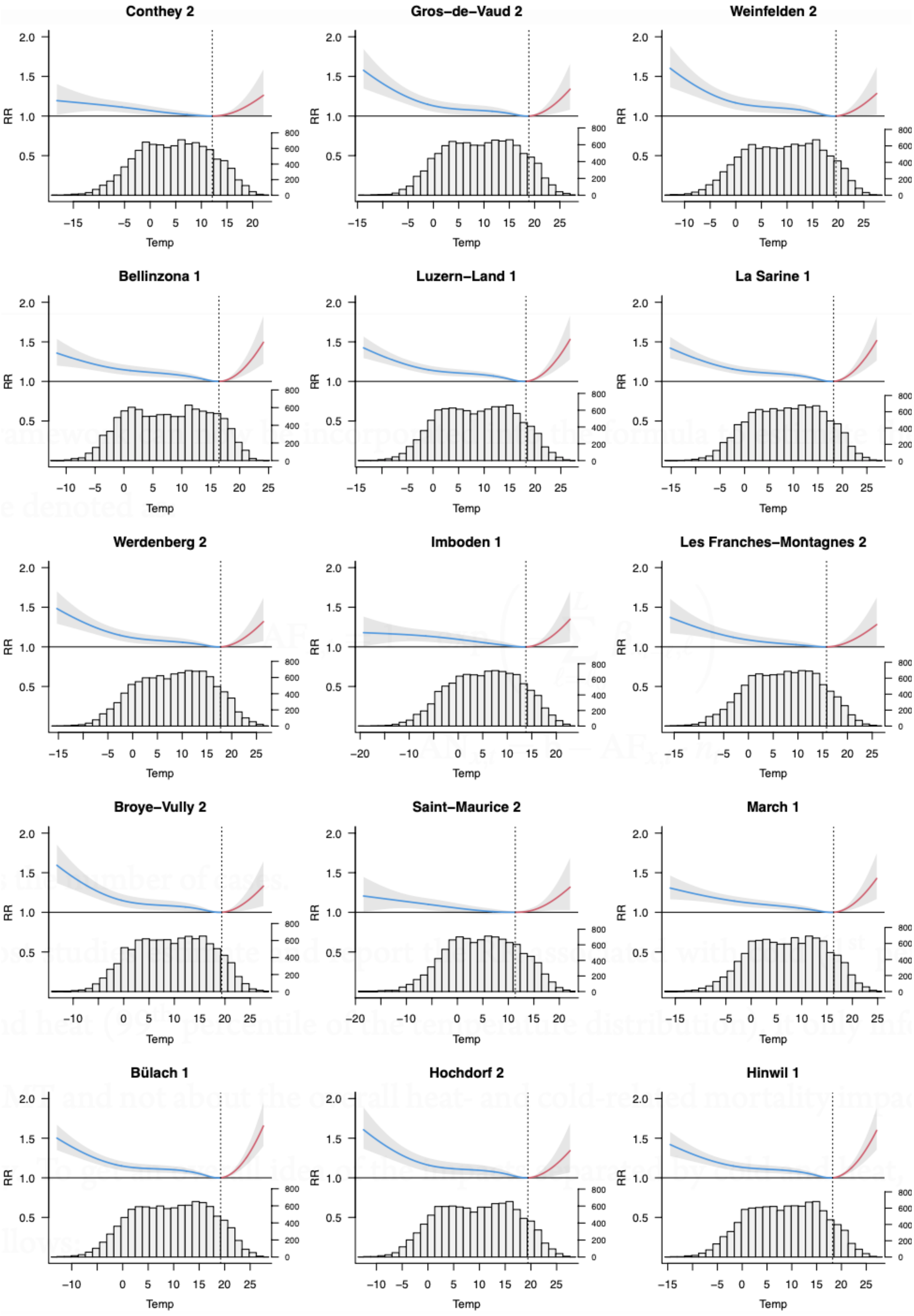
Conthey 2
Gros–de–Vaud 2
Weinfelden 2
Bellinzona 1
Luzern–Land 1
La Sarine 1
Werdenberg 2
Imboden 1
Les Franches–Montagnes 2
Broye–Vully 2
Saint–Maurice 2
March 1
Bülach 1
Hochdorf 2
Hinwil 1
RR
Temp

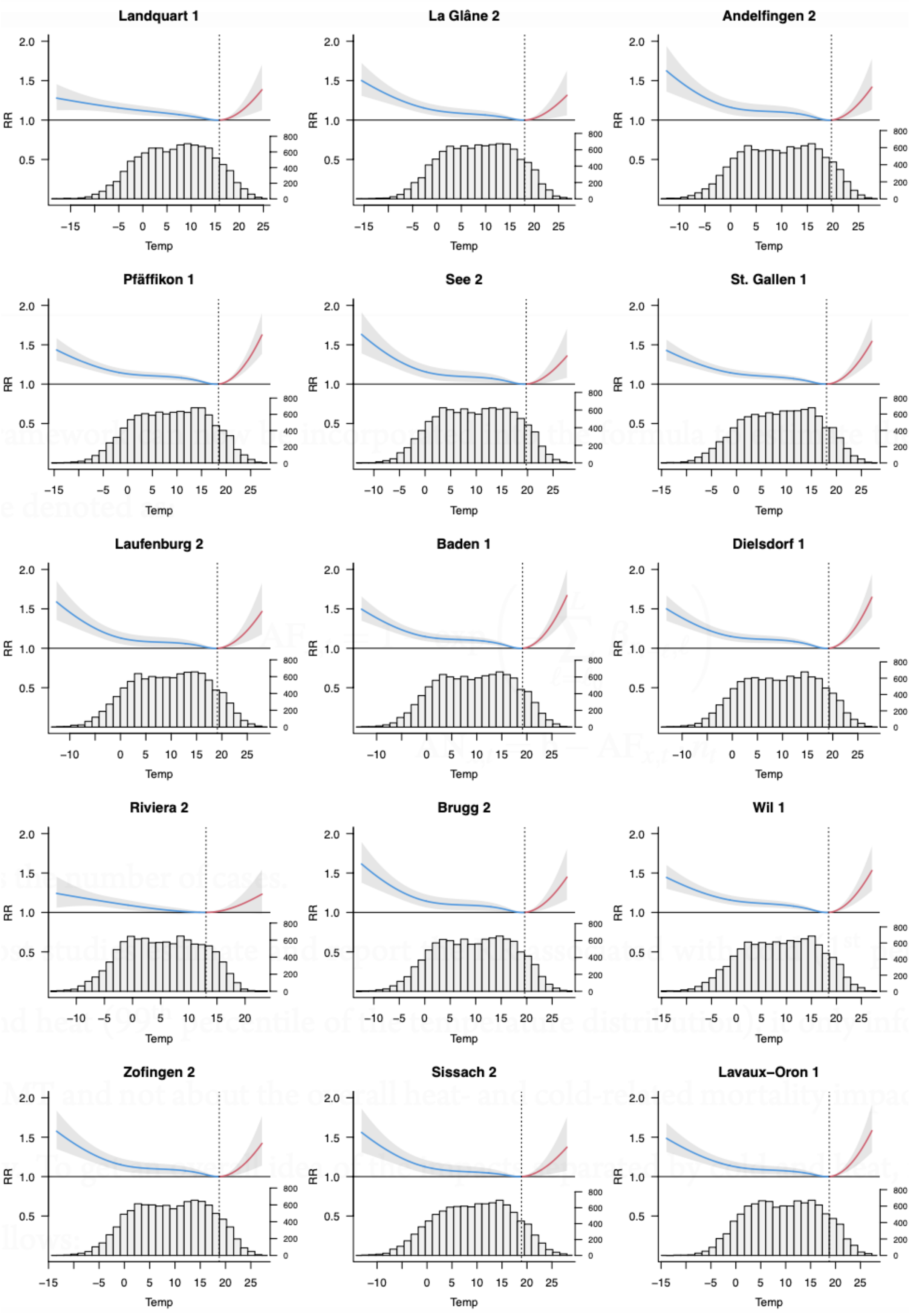
Landquart 1
La Glâne 2
Andelfingen 2
Pfäffikon 1
See 2
St. Gallen 1
Laufenburg 2
Baden 1
Dielsdorf 1
Riviera 2
Brugg 2
Wil 1
Zofingen 2
Sissach 2
Lavaux–Oron 1
RR
Temp

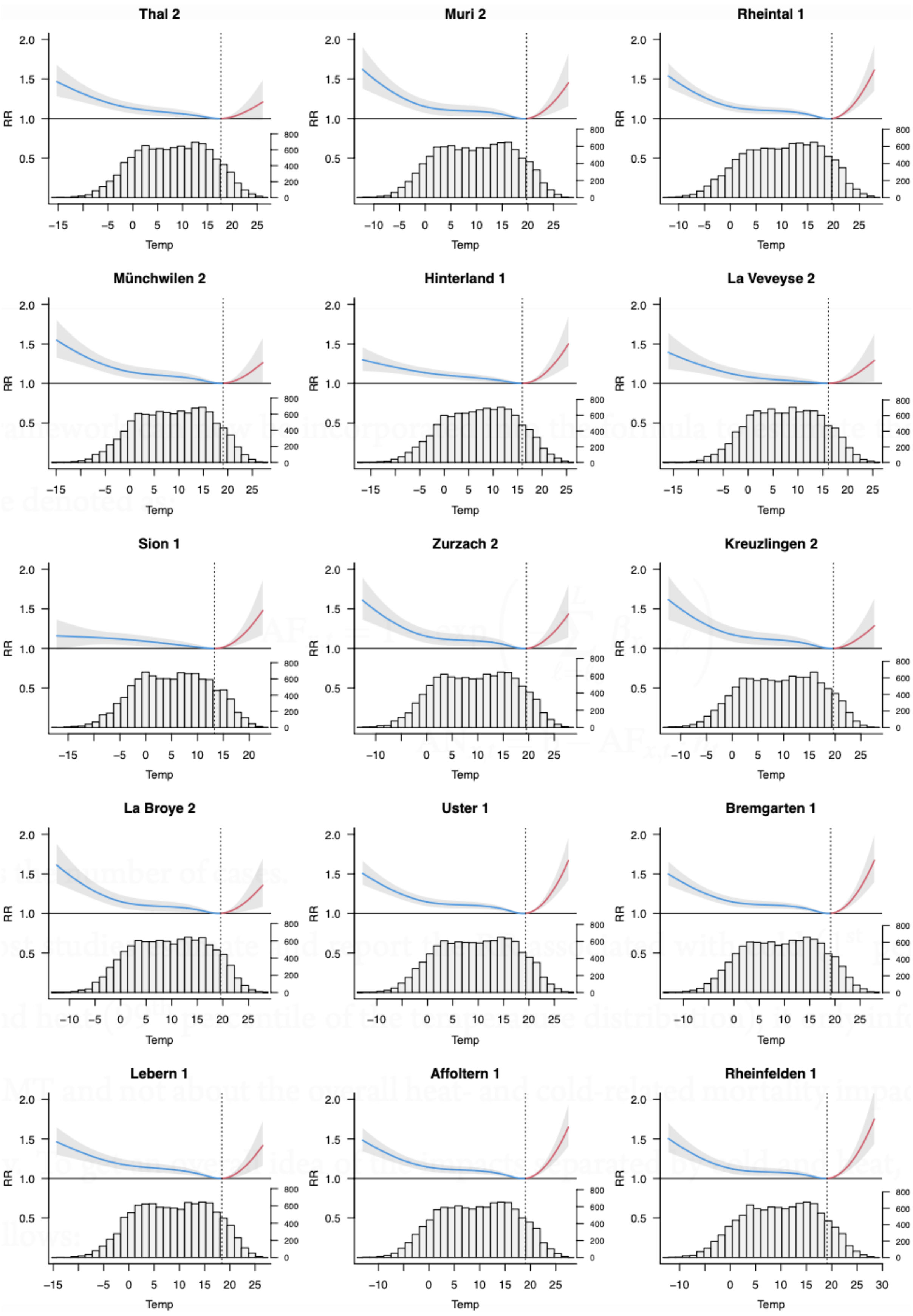

Thal 2
Muri 2
Rheintal 1
Münchwilen 2
Hinterland 1
La Veveyse 2
Sion 1
Zurzach 2
Kreuzlingen 2
La Broye 2
Uster 1
Bremgarten 1
Lebern 1
Affoltern 1
Rheinfelden 1
RR
Temp

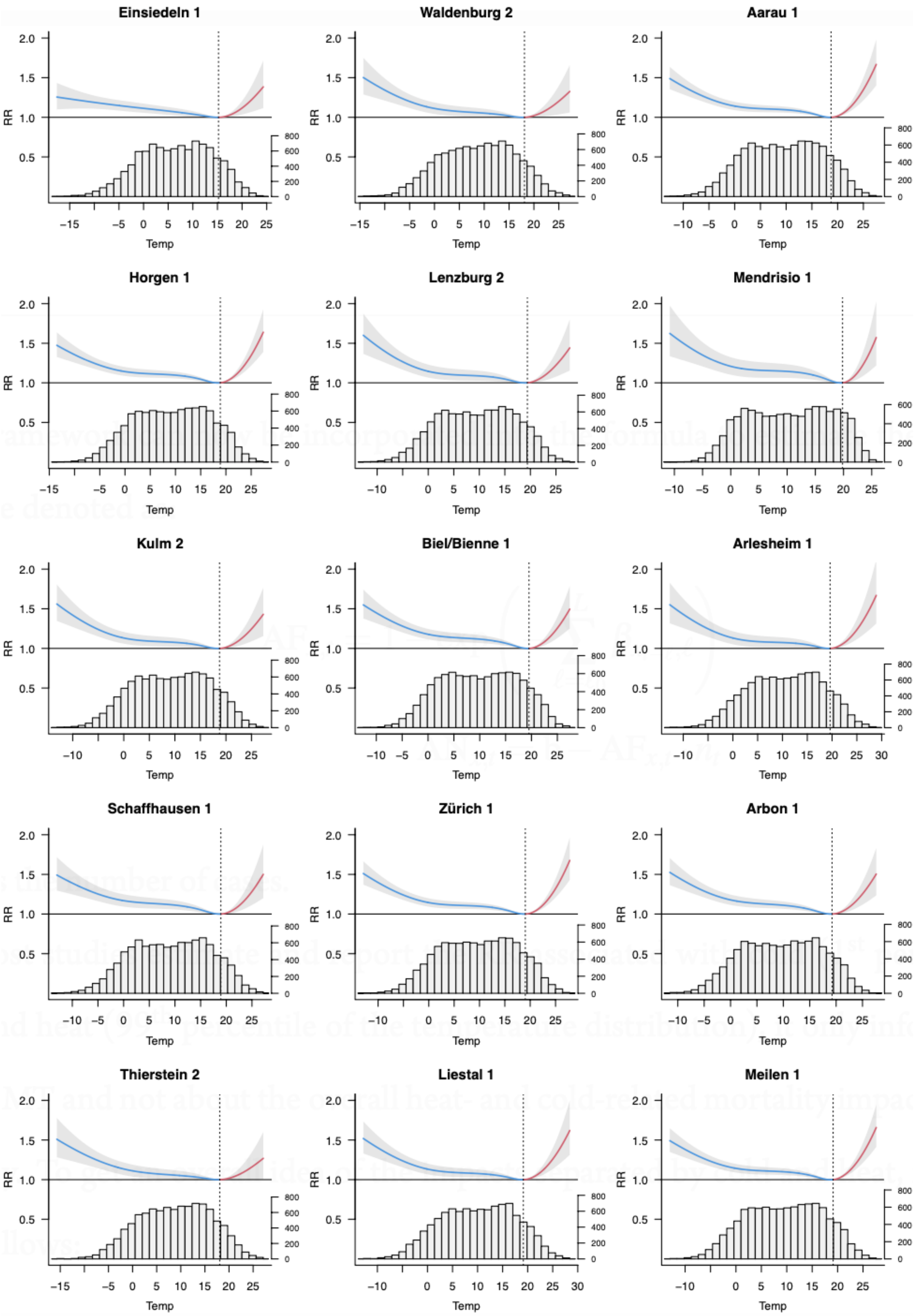

Einsiedeln 1
Waldenburg 2
Aarau 1
Horgen 1
Lenzburg 2
Mendrisio 1
Kulm 2
Biel/Bienne 1
Arlesheim 1
Schaffhausen 1
Zürich 1
Arbon 1
Thierstein 2
Liestal 1
Meilen 1
RR
Temp

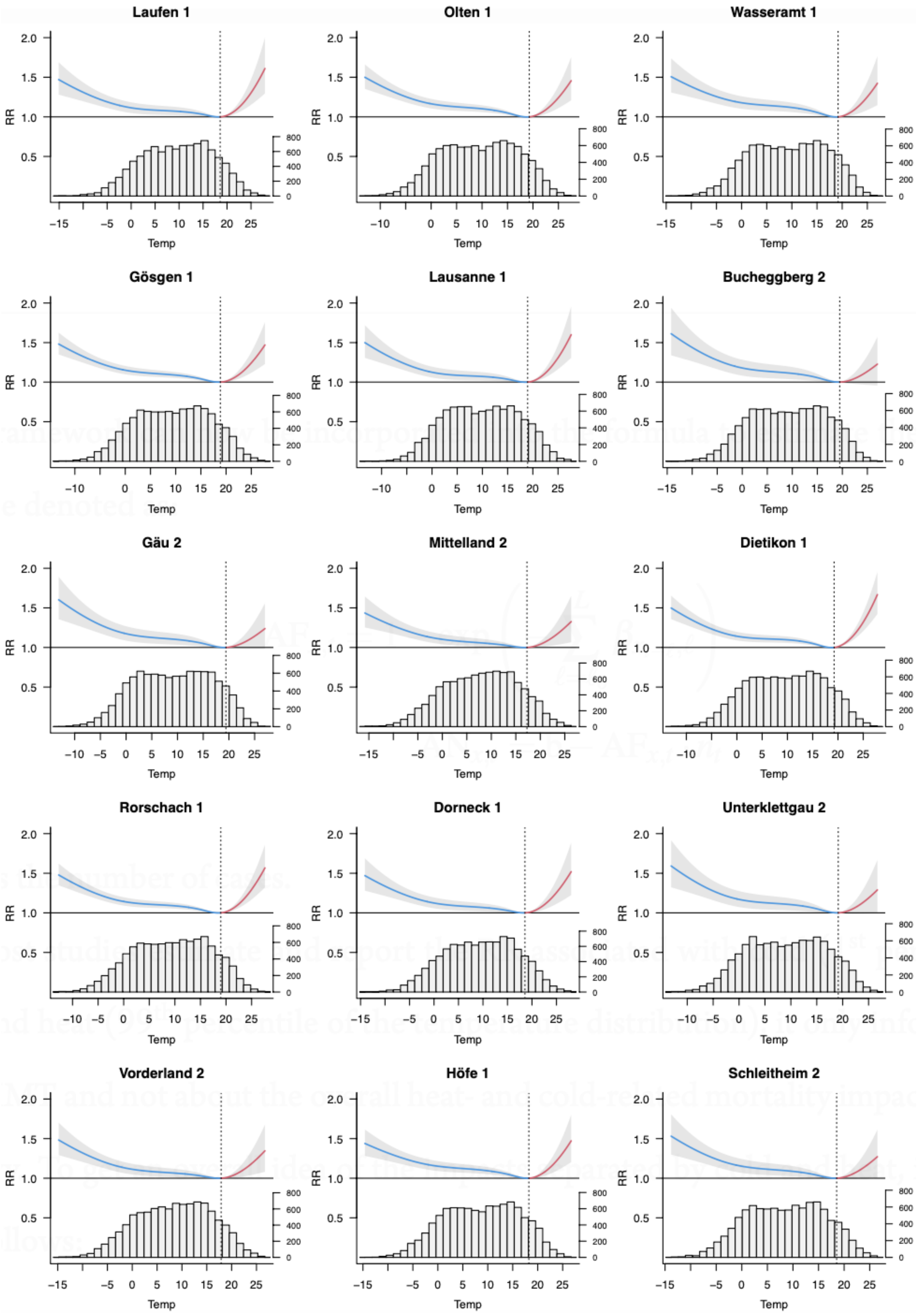
Laufen 1
Olten 1
Wasseramt 1
Gösgen 1
Lausanne 1
Bucheggberg 2
Gäu 2
Mittelland 2
Dietikon 1
Rorschach 1
Dorneck 1
Unterklettgau 2
Vorderland 2
Höfe 1
Schleitheim 2
RR
Temp

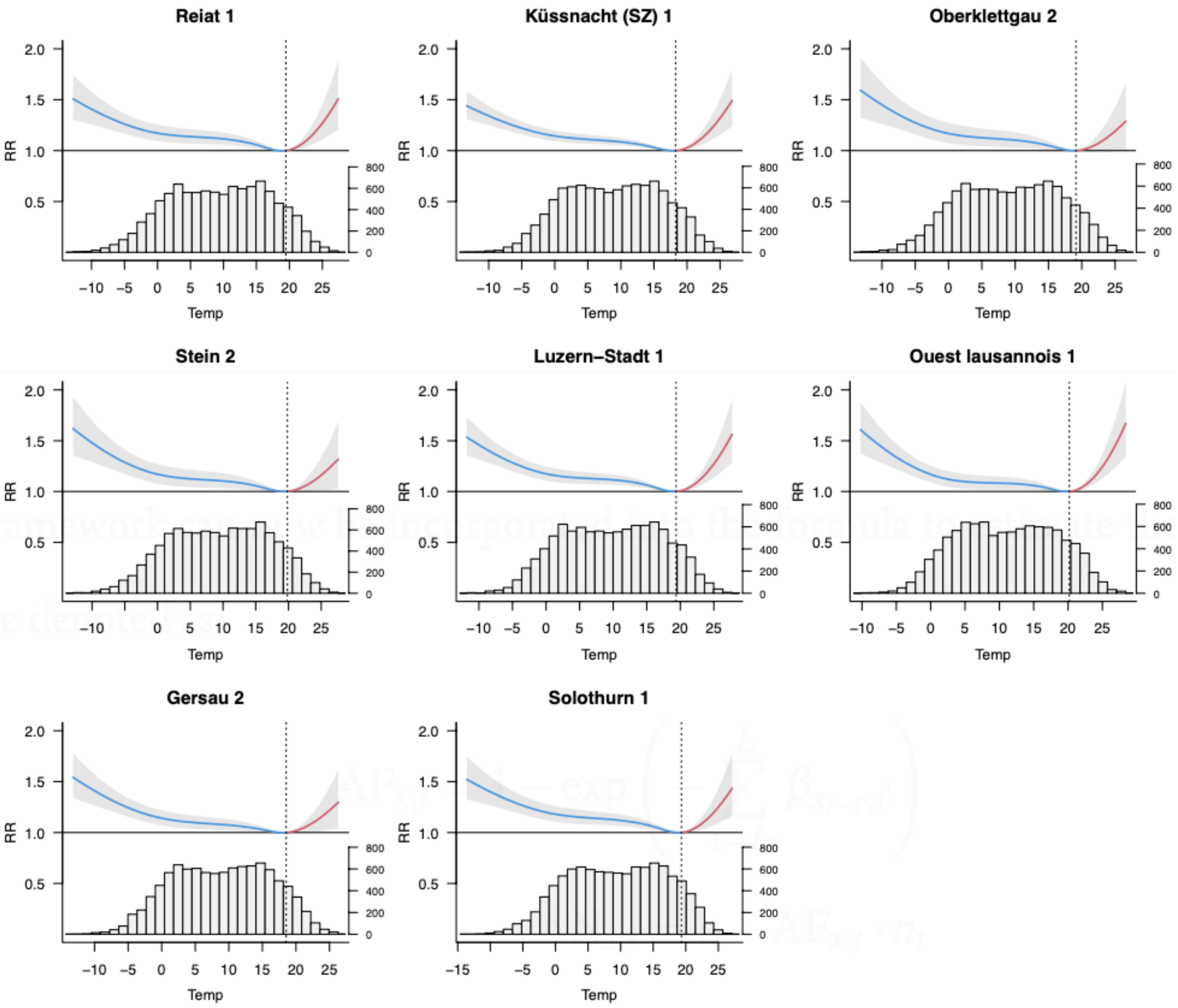
Reiat 1
Küssnacht (SZ) 1
Oberklettgau 2
Stein 2
Luzern–Stadt 1
Ouest lausannois 1
Gersau 2
Solothurn 1
RR
Temp
2.0
1.5
1.0
0.5
800
600
400
200
0
−15
−10
−5
0
5
10
15
20
25

Figure S2. Projected population size, mortality rate and projected annual mortality in Switzerland for SSP2 and SSP5 per 5 year intervals. In blue SSP2 is represented while in red SSP5 is illustrated

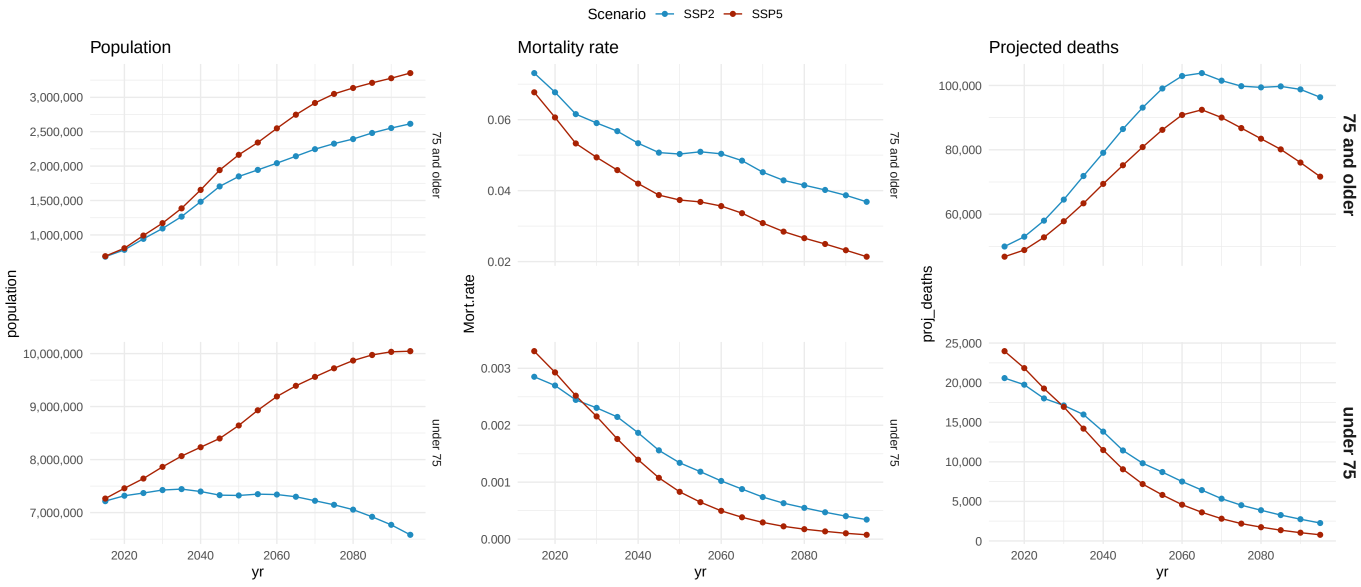

Figure S3. Projected annual mortality (calibrated) compared to the observed annual mortality by age group. In orange, the recalibrated observed annual all-cause mortality is plotted and is connected with the future modelled annual all-cause mortality for SSP2 (green), SSP3 (blue) and SSP5 (Purple)

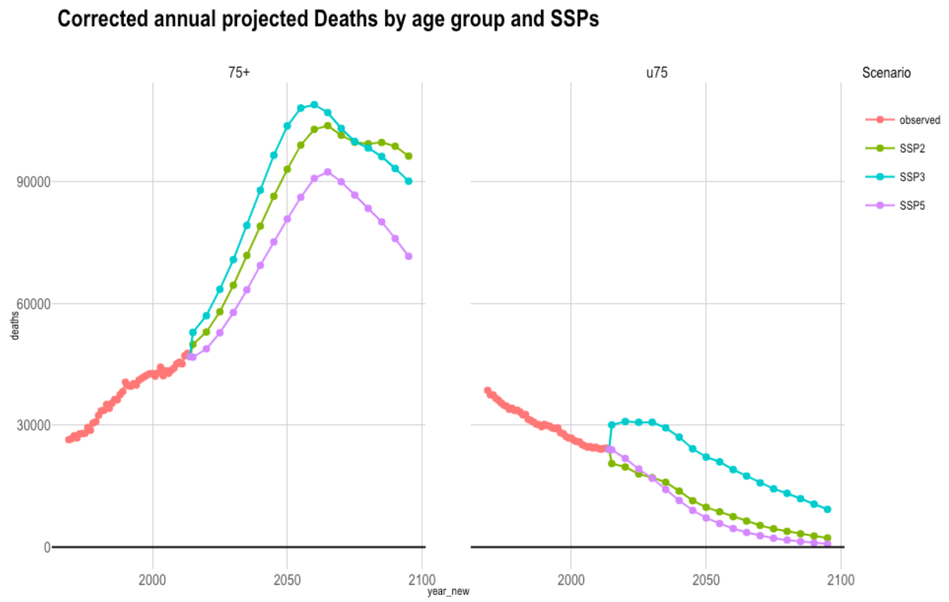

Figure S4. Projected mortality rate for heat- and cold-related mortality impacts under RCP4.5/SSP2 and RCP8.5/SSP5 under 1.5 and 2 and 3 degrees

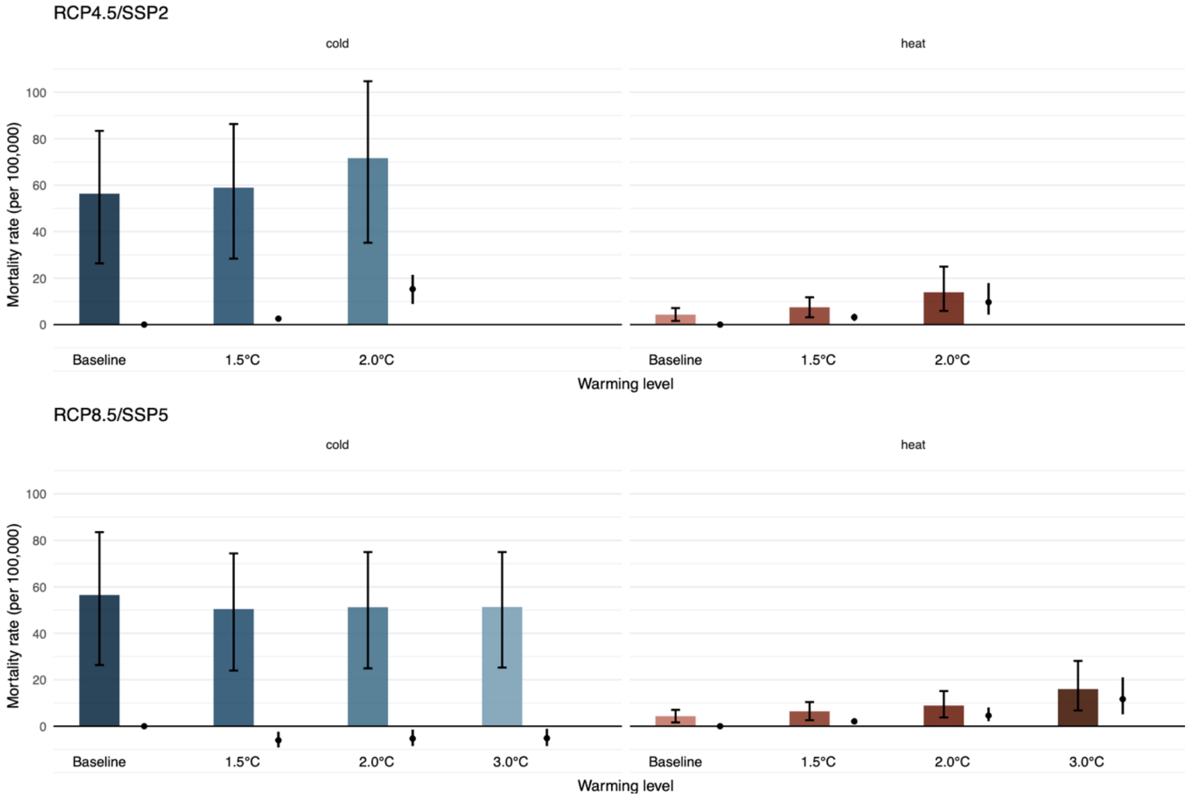

Figure S5. Projected attributable fraction for heat- and cold-related mortality impacts under RCP4.5/SSP2 and RCP8.5/SSP5 under 1.5 and 2 and 3 degrees for each district in Switzerland on a logarithmic scale.

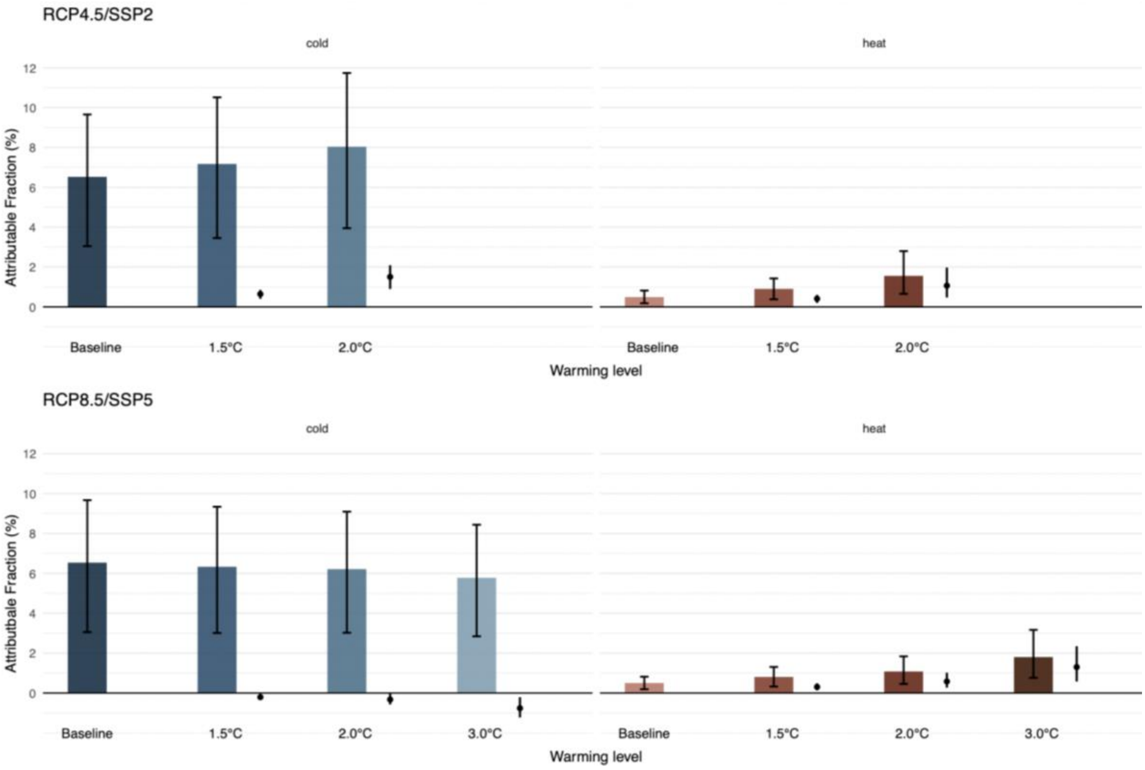
RCP4.5/SSP2
cold
heat
Attributable Fraction (%)
0
2
4
6
8
10
12
Baseline
1.5°C
2.0°C
Warming level
RCP8.5/SSP5
cold
heat
Attributbale Fraction (%)
0
2
4
6
8
10
12
Baseline
1.5°C
2.0°C
3.0°C
Warming level
